# Ultrasmall Gold Nanoparticles (2 nm) Decorated with a High Density of Photochemically Switchable Ligands

**DOI:** 10.1002/chem.202501204

**Published:** 2025-06-01

**Authors:** Lisa‐Sofie Wagner, Tobias Thiele, Kateryna Loza, Christine Beuck, Peter Bayer, Marc Heggen, Michael Giese, Matthias Epple

**Affiliations:** ^1^ Inorganic Chemistry and Center for Nanointegration Duisburg‐Essen (CENIDE) University of Duisburg‐Essen 45141 Essen Germany; ^2^ Organic Chemistry and Center for Nanointegration Duisburg‐Essen (CENIDE) University of Duisburg‐Essen 45141 Essen Germany; ^3^ Structural and Medicinal Biochemistry University of Duisburg‐Essen Universitaetsstr. 2–5 45117 Essen Germany; ^4^ Ernst Ruska Centre for Microscopy and Spectroscopy with Electrons Forschungszentrum Jülich 52428 Jülich Germany

**Keywords:** gold, liquid crystals, nanoparticles, photochemistry, photoswitches

## Abstract

Ultrasmall gold nanoparticles (2 nm) were surface‐coated with photoswitchable 3‐azopyridine ligands by ligand exchange with n‐dodecanethiol‐stabilized gold nanoparticles. Each gold nanoparticle carried about 66 dodecanethiol (DDT) ligands and 49 azo ligands. The azo ligands were reversibly switchable between the stable *E*‐ and the metastable *Z*‐isomer by UV and green light irradiation as shown by UV‐Vis and NMR spectroscopy. The photoswitching was not significantly affected by the conjugation of the azo ligand to the nanoparticle surface, despite the high density of ligands on the particle surface. This offers a pathway for photoswitchable systems on the nanoscale, for example, to manipulate supramolecular systems. The introduction of 0.5 wt% of the nanoparticles into a liquid crystalline host yielded a photoresponsive material which showed a reversible nematic‐to‐isotropic phase transition upon irradiation

## Graphical abstract

Photoswitchable azo ligands can be conjugated to the surface of ultrasmall gold nanoparticles with a high density (about 50 ligands on each nanoparticle). Their photoswitching is not constrained by the binding to the gold surface and can be used to induce a nematic‐to‐isotropic phase transition in a liquid crystal.

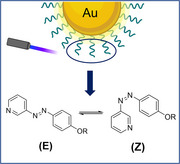

## 1 Introduction

Ultrasmall nanoparticles are on the border between atom‐sharp metal clusters and classical nanoparticles.^[^ ^1^ ^]^ Typically, they have a diameter between 1 and 3 nm. A covalent surface functionalization is possible by attaching ligands during synthesis,^[^ ^2^, ^3^, ^4^, ^5^, ^6^ ^]^ by ligand exchange after synthesis,^[^ ^7^, ^8^, ^9^, ^10^ ^]^ or by subsequent covalent surface modification, for example by click reaction.^[^ ^11^, ^12^, ^13^, ^14^ ^]^

The attachment of photoswitchable ligands to metal nanoparticles has been explored for larger (plasmonic) nanoparticles and for supramolecular systems.^[^ ^15^, ^16^, ^17^, ^18^, ^19^, ^20^ ^]^ This offers a possibility to tune the surface characteristics of a nanoparticle due to folding and unfolding of switchable ligands. Conceptually, this can be used to induce local disorder by switching a ligand back and forth under continuous irradiation. It can also be used to control the arrangement of nanoparticles. The photoswitchable self‐assembly of nanoparticle mesostructures was demonstrated after attaching thiolated azobenzenes onto the surface of 2.5 and 5.5 nm gold nanoparticles (binary mixture)^[^ ^21^ ^]^ or 5.6 nm gold nanoparticles.^[^ ^22^ ^]^ The high number of ligands that can be attached to ultrasmall nanoparticles due to their high curvature (more than 100 on a 2 nm nanoparticle)^[^ ^1^, ^23^ ^]^ leads to a very high local concentration of switchable molecules. The strong gold‐sulfur bond makes the ligand attachment very robust.^[^ ^24^ ^]^ NMR spectroscopy permits to follow the pathway of photoswitching for ultrasmall nanoparticles, in contrast to larger nanoparticles that are not susceptible to NMR spectroscopy.^[^ ^25^, ^26^, ^27^ ^]^ In the context of liquid crystals, larger (usually plasmonic) gold nanoparticles without photoswitches have been proposed as dopant to vary the liquid crystal (LC) properties.^[^ ^28^, ^29^, ^30^, ^31^, ^32^, ^33^, ^34^ ^]^

Here we report a proof‐of‐concept study on the combination of ultrasmall nanoparticles with photoswitchable ligands. We employed these particles to initiate the phase transition of a liquid crystalline material. Photoswitchable 3‐azopyridine ligands were attached to the surface of ultrasmall nanoparticles in a high density. The photoresponse of the 3‐azopyridine was only slightly affected by the attachment to the gold surface, making these particles attractive for the development of photoresponsive hybrid materials or multivalent binding platforms. We demonstrate that the presence of functionalized gold nanoparticles in a liquid crystalline host has only a minor effect on the phase transition temperature but leads to reversible photoresponsive switching from nematic to isotropic upon irradiation with UV light (365 nm).

## 2 Results and Discussion

**Figure 1:**
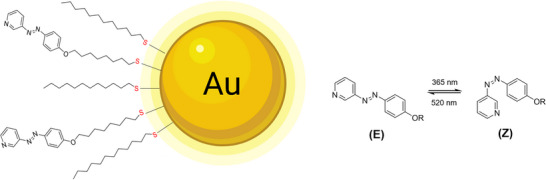
Schematic image of surface‐decorated ultrasmall AuAzo gold nanoparticles, carrying n‐dodecanethiol and the photoswitchable azo ligand **3** (**left**). *E/Z* photoswitching of the ligand **3** (**right**).

The n‐dodecanethiol‐functionalized gold nanoparticles (AuDDT) were synthesized in toluene by a modified Brust–Schiffrin synthesis.^[^ ^35^, ^36^ ^]^ Ligand **3** contained a photoswitchable azopyridine group, together with a spacer and a terminal thiol group to permit covalent attachment to the gold nanoparticle surface. After partial exchange of dodecanethiol (DDT) with ligand **3**, photoswitchable gold nanoparticles (AuAzo) were obtained, which carried both DDT and the photoswitchable ligand **3** on their surface (Figure 1). Ligand **3** can be switched from *E* to *Z* by irradiation with UV‐light (365 nm) and from *Z* to *E* by irradiation with green light (520 nm). All particles were colloidally stable in toluene.

**Figure 2:**
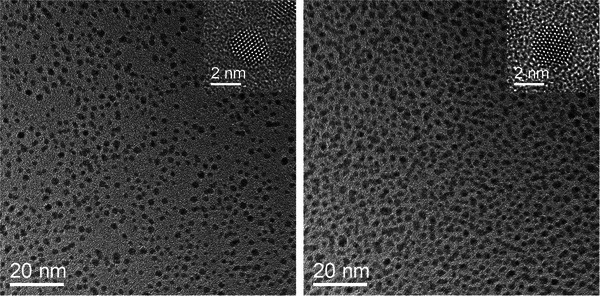
HRTEM images of ultrasmall AuDDT (**left**) and AuAzo (**right**) gold nanoparticles.

The particles were analyzed by complementary methods, including high‐resolution transmission electron microscopy (HRTEM), differential centrifugal sedimentation (DCS), UV‐Vis spectroscopy, and ^1^H NMR spectroscopy in dispersion. Transmission electron microscopy gave average core diameters of 1.8 ± 0.5 nm (AuDDT) and 2.1 ± 0.6 nm (AuAzo). Internally, the gold nanoparticles were well crystalline (Figure 2). As expected, the size and shape of the gold core did not significantly change during the ligand exchange.

**Figure 3:**
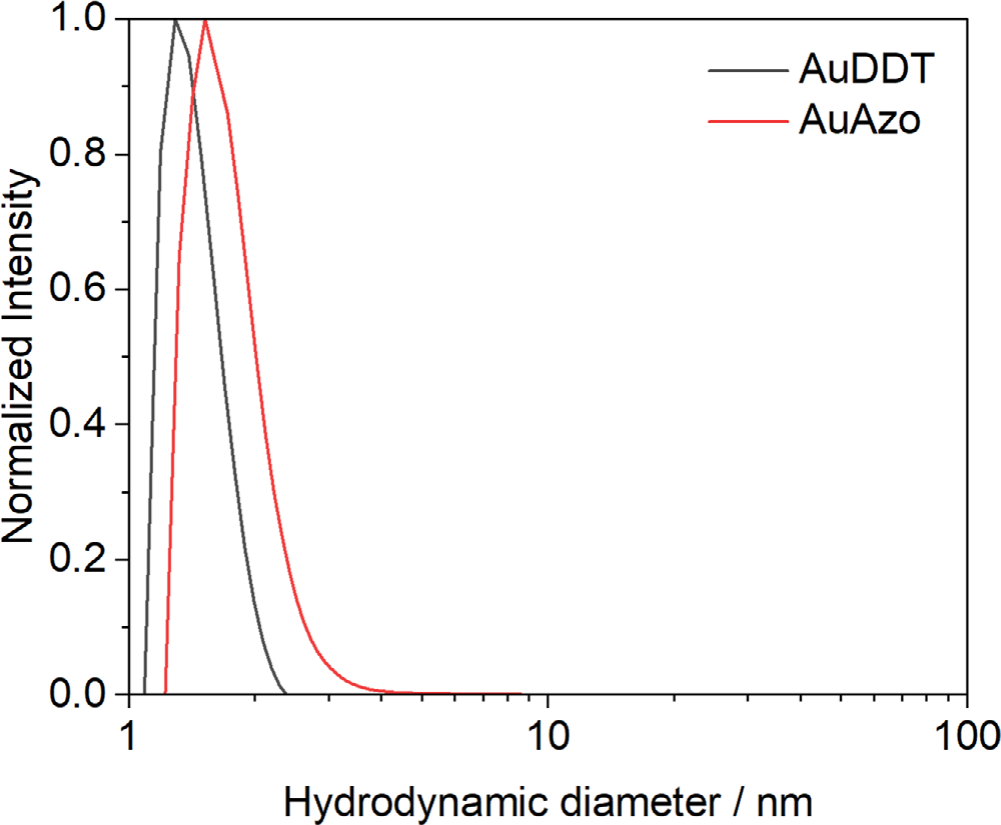
Differential centrifugal sedimentation (DCS) of AuDDT and AuAzo gold nanoparticles, dispersed in toluene. No aggregates or larger particles were present.

The hydrodynamic diameter of the particles was determined by DCS (Figure 3). A monodisperse distribution with no indication for the presence of agglomerates or aggregates was obtained for both particle types. The hydrodynamic diameters of the particles were in the ultrasmall range, with 1.4 ± 0.4 nm for AuDDT nanoparticles and 1.5 ± 0.5 nm for AuAzo nanoparticles. However, DCS generally leads to an underestimation of the diameter for very small particles because the effective density changes due to the ligand shell,^[^ ^37^ ^]^ as reported earlier for similar systems.^[^ ^14^, ^38^ ^]^

**Figure 4:**
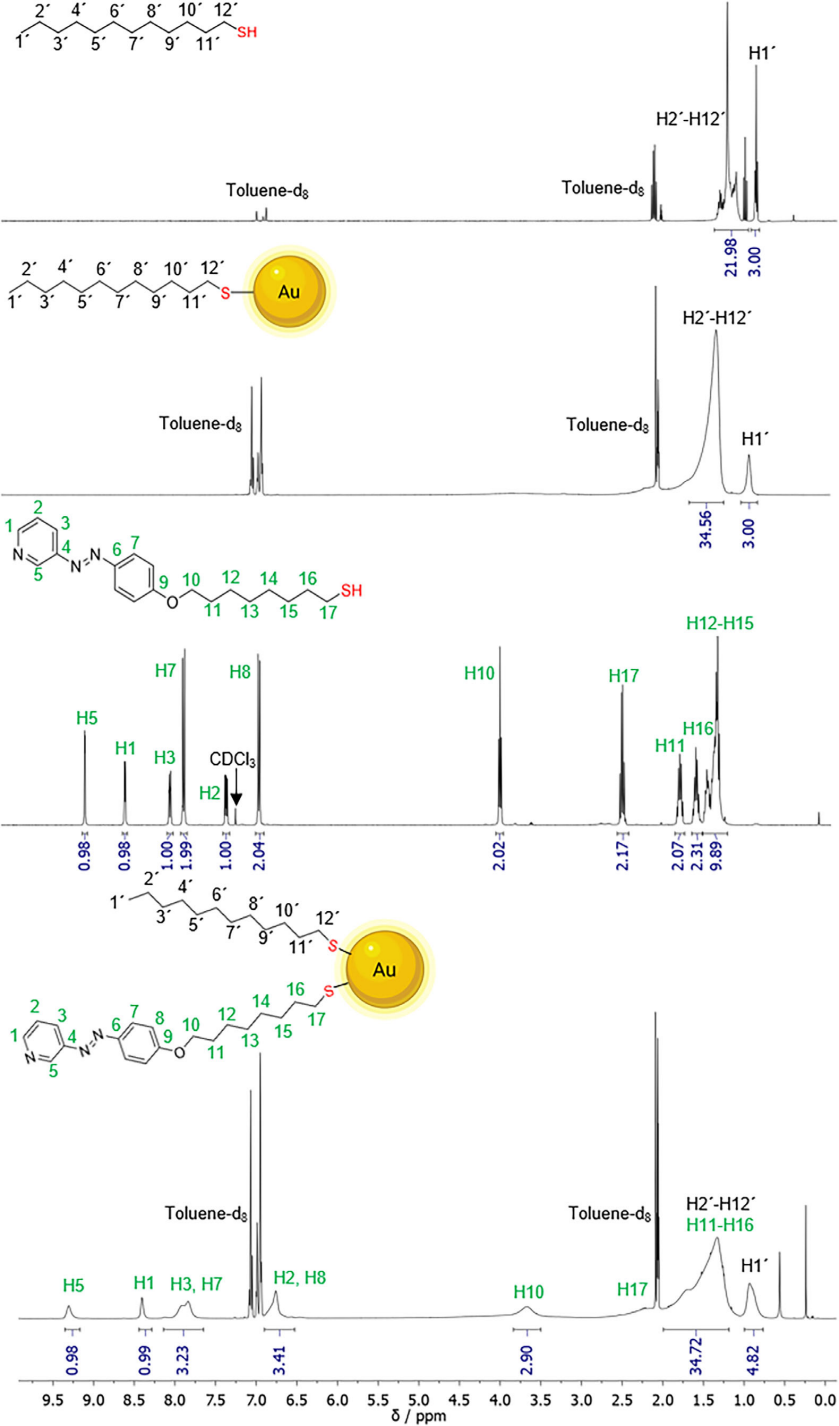
^1^H NMR spectra of the dissolved ligand dodecanethiol (DDT), of AuDDT nanoparticles, of the dissolved ligand **3**, and of AuAzo nanoparticles, all in toluene‐d_8_, except for ligand **3** which was measured in CDCl_3_ (**from top to bottom**).

NMR spectroscopy can be used to confirm the successful attachment of ligands to the nanoparticle surface. This is a viable method for ultrasmall nanoparticles; however, significant peak broadening is always observed, resulting in a considerable decrease in resolution.^[^ ^27^ ^]^ NMR spectroscopy can also be used to determine the ligand ratio on the surface by integration.^[^ ^2^, ^13^, ^38^ ^]^ Figure 4 shows ^1^H‐NMR spectra of free DDT and ligand **3,** as well as ^1^H‐NMR spectra of the nanoparticles AuDDT and AuAzo. As expected, the signals of the ligands on the nanoparticles showed a distinct peak broadening compared to the free ligands.^[^ ^25^, ^26^, ^27^ ^]^ As a result of peak broadening, the multiplicity also disappeared. No additional sharp signals from dissolved ligands were present in the nanoparticle sample, confirming the purity of the nanoparticle dispersion.^[^ ^27^ ^]^

The signal at 0.9 ppm in the spectrum of AuDDT nanoparticles was assigned to the methyl group H1' and the signal at 1.5 ppm to the overlapping methylene groups H2 “‐H12.” In the NMR spectrum of AuAzo nanoparticles, a clear peak broadening was observed as well, demonstrating the successful ligand exchange. The peak broadening was also visible for the aromatic signals between 6.5 ppm and 9.5 ppm, despite the considerable distance of the aromatic protons from the metal core. Usually, the NMR signals become narrower again with increasing distance from the metal core due to the increased mobility.^[^ ^2^ ^]^ By comparison of the spectra of AuDDT and the free ligand **3** with that of AuAzo nanoparticles, the broad signal at 0.9 ppm was assigned to the H1' proton of conjugated DDT. Thus, DDT was still present in the AuAzo nanoparticles, that is, the ligand exchange was only partial (which is not unusual for gold nanoparticles).^[^ ^9^, ^39^ ^]^ To determine the ratio of DDT and ligand **3**, the integrals at 8.4 ppm of ligand **3** (proton H1) and the signal at 0.9 ppm of DDT (protons H1') were normalized to the corresponding number of protons, resulting in a molar ratio of DDT to ligand **3** of about 1.6: 1.

For AuDDT nanoparticles, the number of DDT ligands per particle was 135 as determined by elemental analysis (ratio of sulfur to gold), in good agreement with similar systems. For instance, for glutathione‐functionalized ultrasmall gold nanoparticles (2 nm), 127 ligands were bound to the surface of each nanoparticle.^[^ ^14^ ^]^ With peptide‐functionalized ultrasmall gold nanoparticles, the number of bound ligands was approximately 15 to 150 on each nanoparticle, depending on the length of the peptide chain.^[^ ^2^ ^]^ 135 DDT ligands correspond to a molecular footprint of 0.09 nm^2^ on AuDDT nanoparticles, also in agreement with previous results. The footprint of glutathione on ultrasmall gold nanoparticles was 0.10 nm^2^.^[^ ^14^ ^]^ In comparison with the footprint of peptides of different lengths on ultrasmall gold nanoparticles, the footprint of DDT is similar to that of the dipeptide AlaCys (0.12 nm^2^).^[^ ^2^ ^]^ The tripeptide AlaAlaCys had a footprint of 0.25 nm^2^, and the tripeptide AlaCysAla had a footprint of 0.29 nm^2^.^[^ ^2^ ^]^ Molecular footprints of (16‐mercaptohexadecyl)trimethylammonium bromide (MTAB) on gold nanoparticles of 1.2 to 25 nm were between 0.17 and 0.5 nm^2^.^[^ ^40^ ^]^ For alkanethiols bound to 1.5 to 5.2 nm gold nanoparticles, a footprint of 0.13 to 0.16 nm^2^ was reported.^[^ ^41^ ^]^

The number of ligands per nanoparticle was also determined for AuAzo nanoparticles. As there are now two different ligands on each nanoparticle, at first the mass fraction of sulfur was used to determine the total number of ligands (one sulfur per ligand). This gave 115 ligands per particle. In order to distinguish between DDT and ligand **3**, the mass fraction of nitrogen was used. As nitrogen is only present in ligand **3**, the number of ligand **3** molecules per particle was determined to 49. The number of DDT molecules is the difference between the total number of ligands and the number of ligand **3** molecules, that is, 115–49 = 66 DDT molecules per particle. These ligand numbers result in a ratio of DDT to ligand **3** of 1.4: 1, supporting the results from ^1^H NMR spectroscopy. The fact that the total number of ligands after partial exchange of DDT by ligand **3** is almost constant within the error of the experiment shows that the azo ligand **3** is well integrated into the DDT layer on the gold nanoparticle surface. Details on the computation of ligand concentrations are given in the Experimental Section. Despite the excess of ligand **3**, it was not possible to achieve a full ligand exchange of DDT. However, the only partial exchange may be beneficial for the flexibility of ligand **3** during switching.

**Figure 5:**
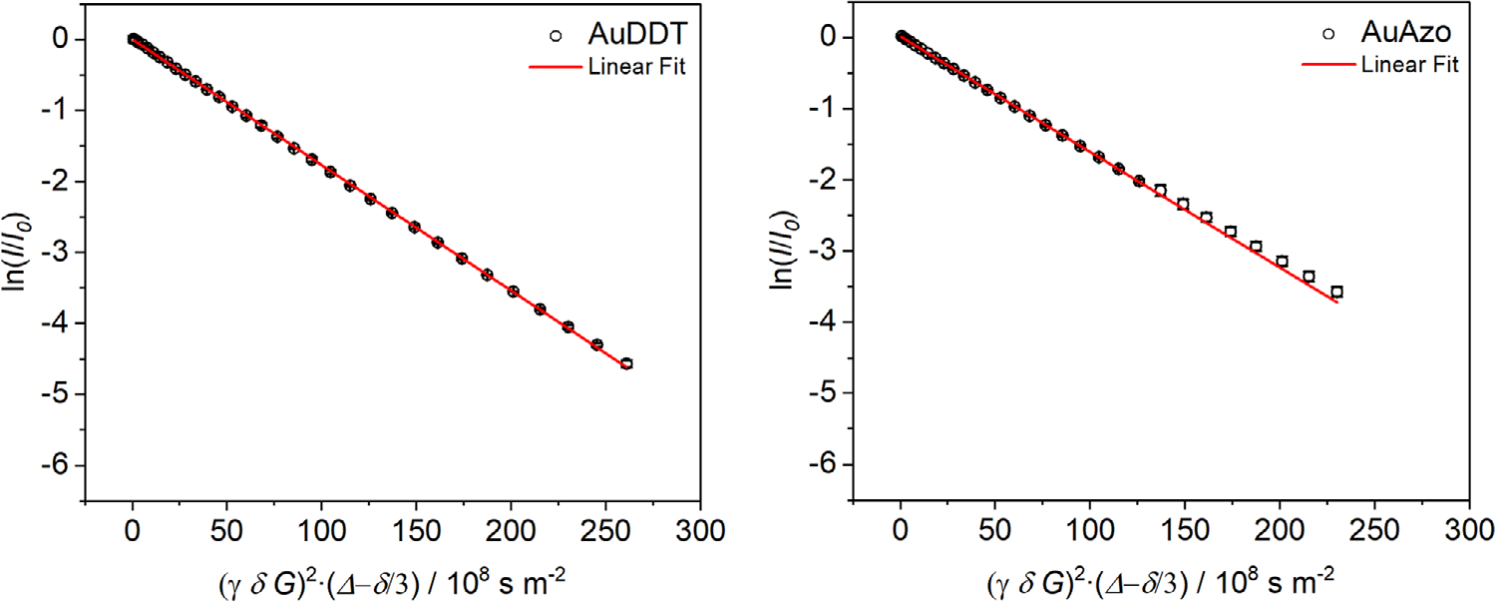
Stejskal–Tanner plots of ^1^H‐DOSY NMR experiments of AuDDT and AuAzo nanoparticles, dispersed in toluene‐d_8_.

The hydrodynamic particle diameter was determined by DOSY NMR spectroscopy. DOSY can also be used to distinguish free unbound ligands from surface‐bound ligands.^[^ ^27^, ^42^ ^]^ Figure 5 shows the Stejskal‐Tanner plot for both particle species.^[^ ^43^ ^]^ By the Stokes‐Einstein equation, the hydrodynamic diameter was computed to 2.8 ± 0.6 nm for AuDDT and 3.1 ± 0.6 nm for AuAzo nanoparticles. The hydrodynamic diameter by DOSY was considerably larger than that obtained by DCS as expected (see above). The difference between the hydrodynamic diameter (about 3 nm) and the metal core diameter (about 2 nm) gives an estimated thickness of the ligand shell of about 0.5 nm, that is, in good agreement with earlier results and similar to the peptide AlaCys.^[^ ^2^ ^]^ Note that the DOSY spectra were measured in toluene whereas DCS was performed in water. The hydrophobic nature of the particles will certainly affect the solvation state of the ligand shell when comparing water and toluene as solvents. All particle characterization data are summarized in Table 1.

**Table 1:** Parameters of AuDDT and AuAzo nanoparticles. Ligand numbers and footprints are based on spherical gold nanoparticles with a diameter of 2 nm.

Nanoparticle type	Hydrodynamic diameter by DCS [nm]	Hydrodynamic diameter by ^1^H‐DOSY NMR [nm]	Core diameter by HRTEM [nm]	Number of ligands	Molecular footprint of ligands [nm^2^]
AuDDT	1.4 ± 0.4	2.8 ± 0.6	1.8 ± 0.5	135 DDT	0.09
AuAzo	1.5 ± 0.5	3.1 ± 0.6	2.1 ± 0.6	66 DDT and 49 ligand **3**	0.11 (average)

**Figure 6:**
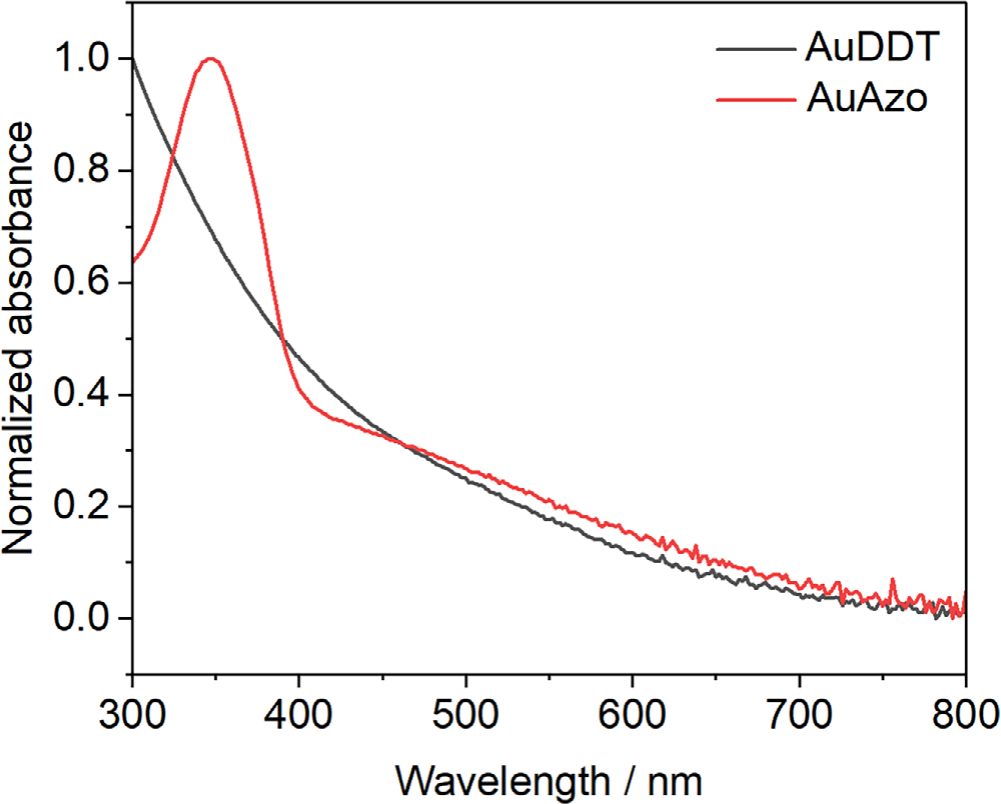
UV‐Vis spectra of ultrasmall AuDDT and AuAzo gold nanoparticles, dispersed in toluene. No plasmon resonance bands were found around 520 nm, indicating the absence of (larger) plasmonic gold nanoparticles. The band at 350 nm is due to the azo group in ligand **3** in AuAzo nanoparticles.

UV‐Vis spectroscopy was used to confirm the absence of plasmonic nanoparticles and the successful attachment of the photoswitchable ligand **3**. Figure 6 shows the UV‐Vis spectra of AuDDT and AuAzo nanoparticles. No surface plasmon resonance (SPR) band was visible around 520 nm, indicating the absence of plasmonic gold nanoparticles larger than about 5 nm.^[^ ^44^, ^45^ ^]^ The UV‐Vis spectrum of AuAzo nanoparticles showed a band corresponding to the azo ligand **3** at 350 nm. In principle, it is possible to determine the number of ligands by quantitative UV spectroscopy.^[^ ^1^ ^]^ However, the azo ligand is photochemically and thermally switchable (*E/Z* transition), therefore its quantification on the particle surface by quantitative UV spectroscopy was not successful as the ratio of *E* and *Z* isomer was not constant, and the measured UV spectrum represents an overlay of both isomers.

The AuAzo nanoparticles were then investigated with respect to their photophysical properties in dispersion, that is, the photoswitching behavior of the azo ligand **3** attached to the nanoparticles was studied. The nanoparticles were dispersed in dichloromethane (DCM) with a maximum absorbance of 0.8 at 350 nm. After taking an initial UV‐Vis spectrum, the sample was irradiated with either 365 nm or 520 nm, respectively, until the photostationary states (PSSs) were reached. Irradiation with 365 nm induces the *E*/*Z* transition (*trans→cis*) whereas irradiation with 520 nm induces the *Z*/*E* transition (*cis→trans*).

**Figure 7:**
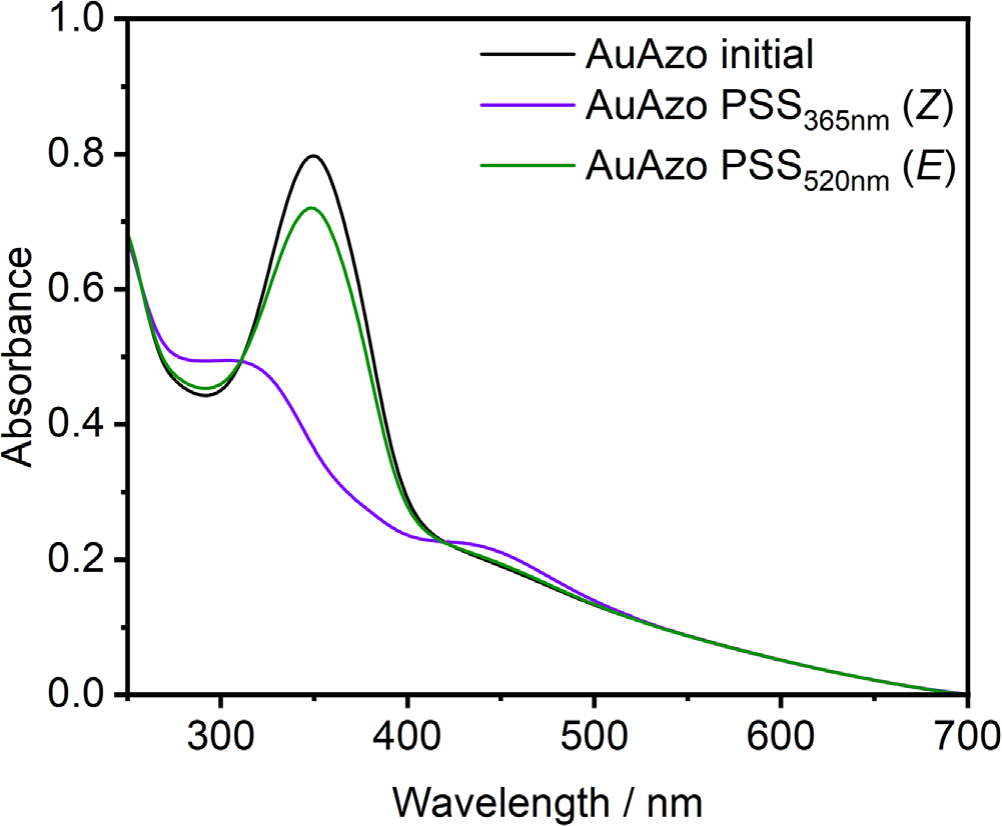
UV‐Vis absorbtion spectra of AuAzo nanoparticles dispersed in DCM. **Black**: Initial spectrum prior to irradiation; **violet**: Spectrum of the photostationary state(PSS) (*Z*) after irradiation with 365 nm for 5 seconds; **green**: Spectrum of the PSSs (*E*) after irradiation with 520 nm for 50 seconds.

Initially a distinct absorption band of the π → π* transition was observed at 350 nm (Figure 7). Upon irradiation with UV‐light (365 nm) the band significantly decreased and shifted to 305 nm, which is characteristic for the *E*/*Z*‐isomerization of azopyridines.^[^ ^46^ ^]^ Simultaneously, an increase in the n → π* band at 430 nm was observed. The PSS was reached after 5 seconds, which was confirmed by further irradiation resulting in an identical spectrum. Afterwards, the sample was irradiated with 520 nm for a *Z*/*E*‐isomerization. 50 second were required to reach the PSS. Indeed, a reverse effect was detected with a lower absorbance at 350 nm compared to the initial spectrum, indicating an incomplete *Z*/*E*‐isomerization, which is consistent with the free ligand **3** (Figure S17). We conclude, however, that the azo ligand **3** can be photoswitched in both directions after covalent conjugation to the gold nanoparticles.

**Figure 8:**
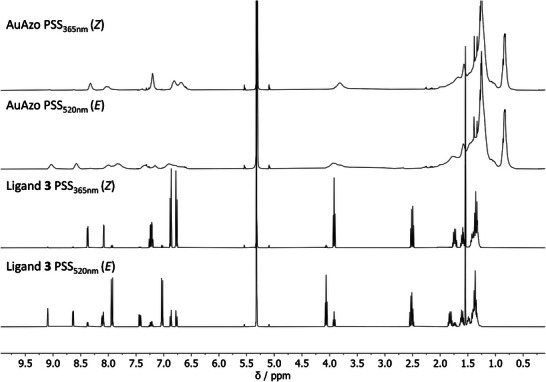
^1^H‐NMR spectra of the dispersed AuAzo nanoparticles and the dissolved ligand **3**, both in DCM‐*d*_2_, for the photostationary states upon irradiation with 365 nm (PSS_365nm_; *Z*) and 520 nm (PSS_520nm_; *E*).

To quantify the photostationary distributions of the *E*‐ and *Z*‐isomers, NMR experiments were conducted. The signals at 8.58 ppm (*E*‐isomer) and 8.33 ppm (*Z*‐isomer), corresponding to proton H1 in ligand **3**, were integrated to determine the *E*/*Z*‐ratio of the azo group. Figure 8 shows the spectra at the PSSs upon irradiation with 365 nm (PSS_365nm_) and 520 nm (PSS_520nm_), respectively, as well as the spectra for the free ligand **3** in both configurations.

After 20 minutes of irradiation of AuAzo nanoparticles with 365 nm, the PSS PSS_365nm_ with ≈ 90% of *Z*‐isomer was observed, confirming that the photoswitching of the azo ligand **3** attached to gold nanoparticles was still possible. On the other hand, the *E*‐rich PSS PSS_520nm_ was reached after 6 hours of irradiation of AuAzo nanoparticles with 520 nm, leading to ≈ 75% *E*‐isomer. These values are in good agreement with those obtained for the dissolved azo ligand **3** (PSS_365nm_ ≈ 94% *Z* after 8 minutes, PSS_520nm_ ≈ 78% *E* after 8 minutes), indicating that the photoswitchability of the azo ligands was not significantly disrupted by steric hinderance after conjugation to the nanoparticles.

We attribute the considerably longer irradiation times for ligand **3** conjugated to the nanoparticles to the absorption by the gold nanoparticles (brown color of the dispersion), reducing the light intensity that reaches the photoswitches. To confirm this, a control experiment was carried out with a mixture of the dissolved azo compound **4** and dispersed AuDDT particles (also having a brown color). Azo compound **4** was chosen instead of ligand **3** to prevent ligand exchange reactions during the experiment. AuDDT and azo compound **4** were dispersed and dissolved in DCM in a molar 1.4: 1 ratio as it is also present in AuAzo nanoparticles for DDT and ligand **3**. In this UV‐Vis photoswitching experiment (Figure S18), the irradiation times to reach the PSS were similar as with the AuAzo nanoparticles. The irradiation times at 520 nm were 50 seconds for AuAzo nanoparticles and 60 seconds for AuDDT + azo compound **4**, indicating that the conjugation of the azo compounds to the nanoparticles was not responsible for the longer irradiation times but rather the light absorption of the inherently brown gold nanoparticles.

**Figure 9:**
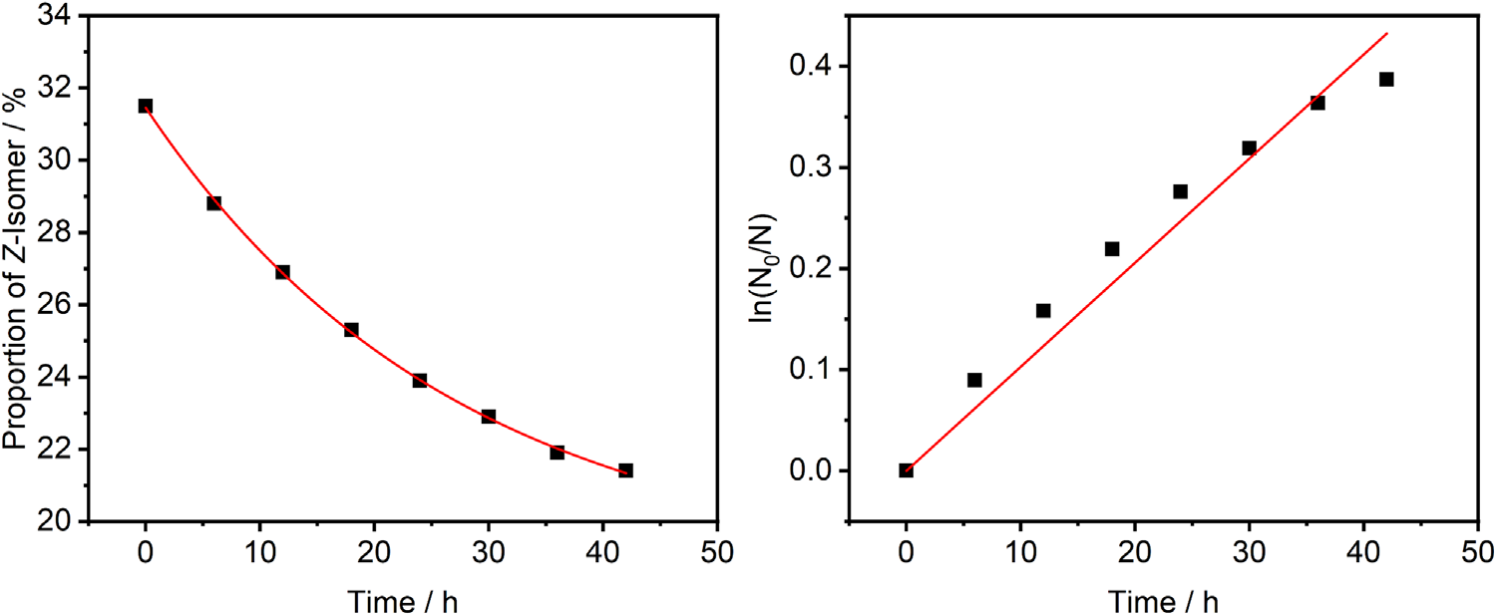
Thermal dark relaxation of AuAzo nanoparticles in DCM‐*d*_2_ at ambient temperature. Plot of the proportion of *Z*‐isomer versus time (**left**) and its linearization (**right**).

The thermal stability of the *Z*‐isomer toward *Z*/*E*‐isomerization was determined by NMR spectroscopy. A sample of AuAzo nanoparticles in DCM‐*d*_2_ was irradiated at 365 nm for several minutes. The sample was then transferred to a brown glass NMR tube and kept in the dark at ambient temperature, while a spectrum was taken every 6 hours. Integration of the same signals as for the PSS experiment and linearization gave the rate constant of the thermal first‐order *Z*/*E*‐isomerization of the azo ligand **3** (Figure 9).

The thermal half‐life time *Z*/*E* of AuAzo nanoparticles was determined as ≈67 hours, which is close to the value of the free ligand **3** (≈59 hours; Figure S19), indicating that the attachment to the nanoparticles did not significantly affect the thermal relaxation of the azo compound. The first‐order rate constant was 0.0103 h^−1^ for the AuAzo nanoparticles and 0.0117 for the dissolved ligand **3**.

**Figure 10:**
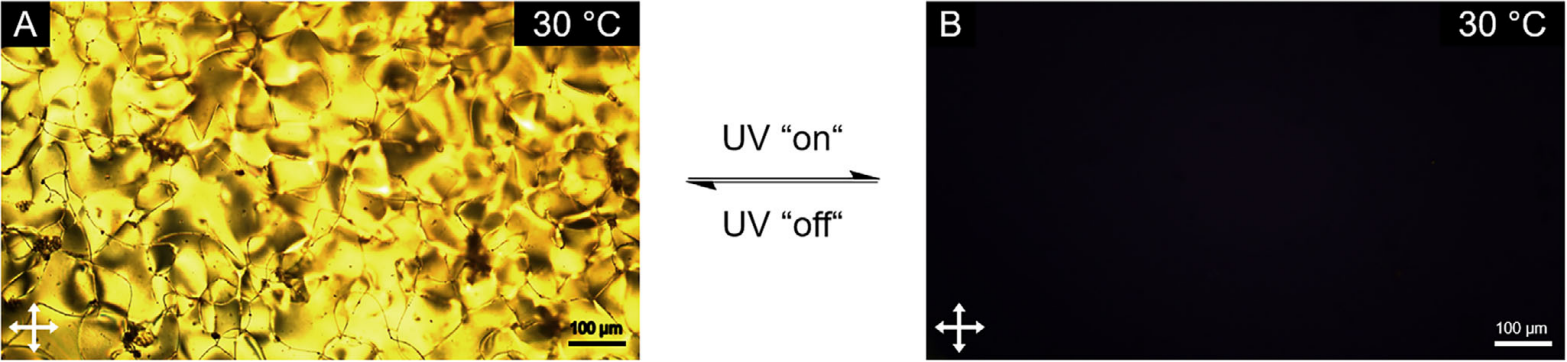
Polarized optical microscopy (POM) images of AuAzo nanoparticles, dispersed in the liquid crystalline host 5CB at 0.5 wt%. The nematic phase was present while the UV light was turned off (**A**, “UV off”), but it turned isotropic phase under UV irradiation (**B** “UV on”). The images were taken under crossed polarizers at 30 °C in the second heating cycle.

Finally, the functionalized nanoparticles were brought into a LC host to study the effect of photoswitching on the orientational supramolecular order in the nematic phase. The AuAzo particles were dispersed in DCM and mixed with the liquid crystalline host 5CB (4‐cyano‐4´‐n‐pentylbiphenyl) phase at 0.5 wt%. After removal of DCM under reduced pressure, the nanoparticle‐doped LC material was obtained, showing a similar phase behavior like the 5CB host.^[^ ^47^ ^]^ Polarized optical microscopy (POM) showed that the nematic‐to‐isotropic phase transition occurred at 33 °C instead of 35 °C for pure 5CB and crystallization was supressed (measured down to 0 °C). A droplet of the material on an untreated glass slide was investigated with respect to the photoresponse of the particles (Figure 10).

Under the POM at 30 °C the materials showed the characteristic texture of the nematic phase (Figure 10A), while irradiation with 365 nm yields an instant extinction of the texture (Figure 10B). The initial texture can be restored by switching off the UV light, that is, the process is reversible. We attribute this behavior to the *E*/*Z* switching and *Z*/*E* relaxation of the azo ligand **3** on the surface of the gold nanoparticles during UV‐light irradiation leading to a disruption of the orientational order in the mesophase. This was confirmed by differential scanning calorimetry (DSC; Figure S20). Similar effects have been reported earlier.^[^ ^48^ ^]^ These results represent a promising start toward functionalized ultrasmall gold nanoparticles in materials applications. The high density of the ligands on the ultrasmall nanoparticles, leading to a very high local concentration in their immediate vicinity inside the LC, probably contributes to this high efficiency.

## 3 Conclusions

Ultrasmall gold nanoparticles with attached photoswitchable ligands can be analyzed by standard procedures for photoswitching experiments, that is, NMR and UV‐Vis spectroscopy. The chemical composition makes the nanoparticles hydrophobic in nature and leads to an excellent dispersibility in organic solvents like DCM or toluene. The photophysical properties are close to those of the dissolved ligand, even at a very high surface loading (49 photoswitchable ligands on each 2 nm nanoparticle). This is favorable for designing photoswitchable particles with tailor‐made properties. Inside a liquid crystalline host material, the nanoparticles induce an instantaneous nematic‐to‐isotropic phase transition under UV‐light irradiation, leading to promising photoresponsive materials.

## 4 Experimental Section

### Reagents

Nitric acid (HNO_3_, 67%), hydrochloric acid (HCl, 37%), and sodium acetate (NaCH_3_COO, analytical grade) were received from Bernd Kraft (Duisburg, Germany). Tetraoctylammonium bromide (>98%), DDT (>98%), sodium borohydride (NaBH_4_, >96%), and 3 kDa spin filters were obtained from Merck (Darmstadt, Germany). Toluene (99.8%), dimethylformamide (DMF, 99.5%), ethanol (99.8%), methanol (99.8%), sodium chloride (NaCl, 99.5%), potassium hydroxide (KOH, analytical grade), and 1,8‐dibromooctane (98%) were obtained from Fisher Scientific (Geel, Belgium). Toluene‐d_8_ (99.5%) was received from Eurisotop (Saarbrücken, Germany). Dichloromethane‐d_2_ (99.6%) and chloroform‐d (99.8%) were obtained from deutero (Kastellaun, Germany). Chloroform‐d was filtered over aluminum oxide before use. Acetonitrile (HPLC grade), hydrochloric acid (HCl, 37%), and acetic acid (glacial) were obtained from VWR (Dietikon, Switzerland). Potassium thioacetate (97%), 4‐cyano‐4´‐n‐pentylbiphenyl (98%) and 3‐aminopyridine (99%), were obtained from TCI (Tokyo, Japan). Magnesium sulfate (99%) and potassium carbonate (99%) were received from Carl Roth (Karlsruhe, Germany). Phenol (99%) and octyl bromide (98%) were obtained from abcr (Karlsruhe, Germany). Sodium nitrite was received from BASF (Ludwigshafen am Rhein, Germany). Cyclohexane and ethyl acetate were obtained from Caldic (Düsseldorf, Germany) in technical purity and distilled before use.

Ultrapure water (Purelab Ultra instrument, 18.2 MΩ, ELGA) was used. All glassware for nanoparticle synthesis was cleaned by boiling once with concentrated nitric acid (67% in water), followed by rinsing with water. Silica gel (60 M, 0.04 – 0.063 mm) was received from Machery Nagel (Düren, Germany).

### Synthesis of the ligand AzoAlkyl 3


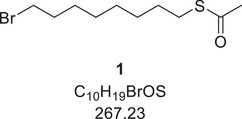


Alkylation agent **1** was synthesized according to the literature.^[^ ^49^ ^]^ 1.91 g (16.7 mmol, 1.0 eq.) potassium thioacetate and 10 mL (14.6 g, 53.7 mmol, 3.2 eq.) 1,8‐dibromooctane were dissolved in 150 mL acetonitrile and refluxed overnight. The salts were filtered off and the solvent was removed under reduced pressure. The crude product was purified by flash column chromatography on silica gel with a gradient of pure cyclohexane to 95:5 cyclohexane:ethyl acetate as eluent. The product (3.50 g, 13.1 mmol, 78%) was obtained as a colorless oil.

^1^H‐NMR (400 MHz, CDCl_3_, 298 K): *δ* [ppm] = 3.40 (t, ^3^ *J*  =  6.8 Hz, 2 H), 2.86 (t, ^3^ *J*  =  7.4 Hz, 2 H), 2.32 (s, 3 H), 1.85 (m, 2 H), 1.55 (m, 2 H), 1.47–1.26 (m, 8 H).


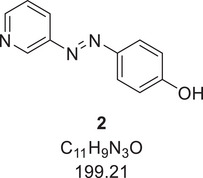


Azo compound **2** was synthesized according to the literature.^[^ ^15^ ^]^ 4.92 g (52.3 mmol, 1.0 eq.) 3‐aminopyridine was suspended in 75 mL glacial acetic acid and 15 mL hydrochloric acid (12 M) and cooled to 0 °C. 4.03 g (58.4 mmol, 1.1 eq.) sodium nitrite was dissolved in a minimum amount of water and added dropwise under stirring for 30 minutes. Separately, 7.06 g (75.0 mmol, 1.4 eq.) phenol and 22.5 g (166 mmol, 3.2 eq.) sodium acetate were dissolved in a mixture of 50 mL ethanol and 50 mL water and added to the diazonium salt. After stirring for one hour, the resulting precipitate was isolated by filtration, washed with water, and dried *in vacuo*. The product was obtained as an orange solid (7.98 g, 40.0 mmol, 77%).

^1^H‐NMR (400 MHz, DMSO‐*d*_6_, 298 K): *δ* [ppm] = 10.51 (s (br), 1 H), 9.03 (d, ^4^ *J*  =  2.4 Hz, 1 H), 8.67 (dd, ^3^ *J*  =  4.7, ^4^ *J*  =  1.6 Hz, 1 H), 8.10 (ddd, ^3^ *J*  =  8.2, ^4^ *J*  =  2.4, 1.6 Hz, 1 H), 7.84 (m, 2 H), 7.58 (dd, ^3^ *J*  =  8.2, 4.7 Hz, 1 H).


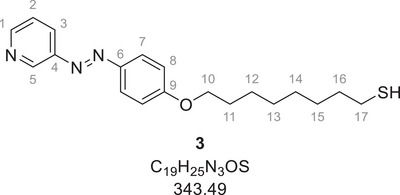


Ligand **3** was synthesized according to the literature.^[^ ^50^ ^]^ 1.90 g (7.12 mmol, 1.0 eq.) alkylation agent **1**, 1.60 g (8.01 mmol, 1.1 eq.) azo compound **2** and 1.65 g (11.9 mmol, 1.7 eq.) potassium carbonate were dissolved in 170 mL acetonitrile and refluxed overnight. After removing the solvent under reduced pressure, 150 mL ethanol and 2.51 g (44.7 mmol, 6.3 eq.) potassium hydroxide dissolved in 20 mL water were added. The reaction mixture was refluxed for 4 hours. After cooling to room temperature, 70 mL of 1 M hydrochloric acid was added and the solvent was removed under reduced pressure. The crude product was adsorbed onto celite and purified by flash column chromatography on silica gel with 2:1 cyclohexane:ethyl acetate as eluent. The product (519 mg, 1.51 mmol, 21%) was obtained as an orange solid.

Melting point: 81 °C

^1^H‐NMR (400 MHz, CDCl_3_, 298 K): *δ* [ppm] = 9.12 (d, ^4^ *J*  =  2.2 Hz, 1 H, C^5^─H), 8.62 (dd, ^3^ *J*  =  4.8, ^4^ *J*  = 1.4 Hz, 1 H, C^1^─H), 8.07 (ddd, ^3^ *J*  =  8.2, ^4^ *J*  =  2.2 Hz, 1 H, C^3^─H), 7.90 (d, ^3^ *J*  =  9.0 Hz, 2 H, C^7^─H), 7.38 (dd, ^3^ *J*  =  8.2, 4.8 Hz, 1 H, C^2^─H), 6.97 (d, ^3^ *J*  =  9.0 Hz, 2 H, C^8^─H), 4.00 (t, ^3^ *J*  =  6.5 Hz, 2 H, C^10^─H_2_), 2.49 (dt, ^3^ *J*  =  7.4, 7.3 Hz, 2 H, C^17^─H_2_), 1.77 (m, 2 H, C^11^─H_2_), 1.57 (m, 2 H, C^16^─H_2_), 1.49^–^1.25 (m, 8 H, C^12–15^─H_2_).

^13^C‐NMR (101 MHz, CDCl_3_, 298 K): *δ* [ppm]  = 162.32 (C^9^), 150.97 (C^1^), 148.02 (C^4^), 147.02 (C^5^), 146.82 (C^6^), 126.77 (C^3^), 125.14 (C^7^), 123.93 (C^2^), 114.80 (C^8^), 68.37 (C^10^), 33.99 (C^16^), 29.25 (C^11–15^), 29.14 (C^11–15^), 29.00 (C^11–15^), 28.29 (C^11–15^), 25.96 (C^11–15^), 24.64 (C^17^).

HR‐MS (ESI‐pos., MeOH), *m*/*z*: calc. for [C_19_H_25_N_3_OS+H]^+^: 344.1791; found: 344.1812; calc. for [C_19_H_25_N_3_OS+Na]^+^: 366.1611; found: 366.1615.

IR (ATR), *ṽ* [cm^−1^]: 2919, 2850, 1601, 1580, 1499, 1466, 1417, 1388, 1323, 1299, 1250, 1139, 1107, 1016, 841, 813, 722, 698, 553.


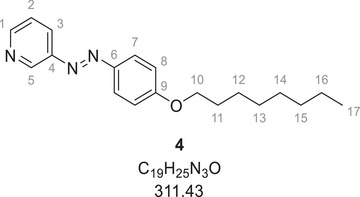


Compound **4** was synthesized according to the literature.^[^ ^51^ ^]^ 720 mg (3.61 mmol, 1.0 eq.) azo compound **2**, 783 mg (5.67 mmol, 1.6 eq.) potassium carbonate and 0.75 mL (833 mg, 4.31 mmol, 1.2 eq.) octylbromide were suspended in 10 mL DMF and stirred at 120 °C overnight. After cooling to room temperature, 150 mL brine (saturated aqueous sodium chloride solution) was added and extracted twice with ethyl acetate. The organic layers were washed with brine again, dried over magnesium sulfate, filtrated and concentrated under reduced pressure. The crude product was purified by flash column chromatography on silica gel with 5:3 cyclohexane:ethyl acetate as eluent. The product (888 mg, 2.85 mmol, 79%) was obtained as an orange solid.

Melting point: 83 °C

^1^H‐NMR (400 MHz, CDCl_3_, 298 K): *δ* [ppm] = 9.14 (dd, ^4^ *J*  =  2.2 Hz, 1 H, C^5^─H), 8.66 (dd, ^3^ *J*  =  4.8, ^4^ *J*  =  1.5 Hz, 1 H, C^1^─H), 8.12 (ddd, ^3^ *J*  =  8.2 Hz, ^4^ *J*  = 2.2 Hz, 1 H, C^3^─H), 7.94 (d, ^3^ *J*  =  9.0 Hz, 2 H, C^7^─H), 7.43 (ddd, ^3^ *J*  =  8.2, 4.8 Hz, 1 H, C^2^─H), 7.02 (d, ^3^ *J*  =  9.0 Hz, 2 H, C^8^─H), 4.05 (t, ^3^ *J*  =  6.5 Hz, 2 H, C^10^─H_2_), 1.82 (m, 2 H, C^11^─H_2_), 1.48 (m, 2 H, C^12^─H_2_), 1.41–1.24 (m, 8 H, C^13–16^─H_2_), 0.89 (t, ^3^ *J*  =  6.8 Hz, 3 H, C^17^─H_3_).

^13^C‐NMR (101 MHz, CDCl_3_, 298 K) *δ* [ppm]  = 162.54 (C^9^), 150.87 (C^1^), 148.24 (C^4^), 146.95 (C^5^), 146.93 (C^6^), 127.12 (C^3^), 125.99 (C^7^), 124.11 (C^2^), 114.96 (C^8^), 68.61 (C^10^), 31.95 (C^16^), 29.48 (C^13/14^), 29.37 (C^13/14^), 29.30 (C^11^), 26.15 (C^12^), 22.80 (C^15^), 14.24 (C^17^).

HR‐MS (ESI‐pos., MeOH), *m*/*z*: calc. for [C_19_H_25_N_3_O+H]^+^: 312.2070; found: 312.2075.

IR (ATR), *ṽ* [cm^−1^]: 2918, 2851, 1599, 1580, 1498, 1466, 1420, 1383, 1322, 1297, 1252, 1138, 1108, 1044, 1020, 997, 843, 812, 721, 698, 552, 494.

Elemental analysis (CHN) [%]: calc. for C_19_H_25_N_3_O: C 73.28, H 8.09, N 13.49; found: C 73.65, H 8.08, N 13.35.

### Synthesis of DDT‐capped gold nanoparticles (AuDDT)

The synthesis was carried out with a modified Brust–Schiffrin synthesis.^[^ ^35^, ^52^ ^]^ Tetrachloroauric acid (4 mg Au, 20 µmol) was dissolved in 30 mL degassed water and 30 mL toluene. To transfer the gold to the organic phase, tetraoctylammonium bromide (2.5 eq., 16 mg, 50 µmol) dissolved in 1 mL toluene was used as a phase transfer catalyst. After stirring for 10 minutes, the organic phase was separated from the aqueous phase, and DDT (2.5 eq., 11.8 µL, 50 µmol) was added rapidly. The solution was stirred for 20 minutes, during which the initially yellow solution turned colorless. Then, a freshly prepared solution of sodium borohydride in ice‐cold water (7.5 eq., 5.7 mg, 150 µmol, 7.6 g L^−1^) was quickly added. The solution was stirred for another 30 minutes. The particles were purified by precipitation. For this, the reaction solution was diluted to 400 mL with methanol and left without stirring for 12 hours at −20 °C. The precipitated AuDDT nanoparticles were isolated at 4000 rpm (2500 g). Afterwards, they were redispersed in 8 mL toluene and again diluted with 400 mL methanol and stored at −20 °C for 12 hours. This procedure was repeated four times. The dispersed nanoparticles had a brown color.

### Synthesis of azopyridine ligand‐functionalized gold nanoparticles (AuAzo)

The synthesis was accomplished via ligand exchange of DDT by ligand **3**. For this, AuDDT nanoparticles (4 mg Au, 20 µmol) were dispersed in 20 mL toluene. Subsequently, ligand **3** (4.35 eq., 30 mg, 87 µmol) dissolved in 1 mL of toluene was added. The mixture was stirred for 72 hours at room temperature under light exclusion. Purification was performed in the same way as for AuDDT nanoparticles. The dispersed nanoparticles had a brown color.

### Computation of the ratio of ligands to nanoparticles

The average number of ligands on each nanoparticle was estimated by ICP‐MS elemental analysis, using the molar ratios of sulfur to gold and to nitrogen. This is outlined exemplarily in the following for AuDDT nanoparticles. To obtain the amounts of substance of the elements, the obtained mass fractions *w* were multiplied by the total mass of the sample:

(1) $$\;n_{\mathrm{element}}=\frac{m_{\mathrm{total}\;}\cdot w_{\mathrm{element}}}{M_{\mathrm{element}}}$$

For AuDDT nanoparticles, this resulted in the amounts of substances for gold and sulfur:

(2) $$\;n_{\mathrm{Au}}=\;\frac{1\;g\cdot 5.55\cdot 10^{-3}}{196.97\frac{g}{mol}}=2.82\;\cdot 10^{-5}\;\mathrm{mol}$$

(3) $$\;n_{S}=\;\frac{1\;g\cdot 4.92\cdot 10^{-4}}{32.07\frac{g}{mol}}=1.53\;\cdot 10^{-5}\;\mathrm{mol}$$

The molar ratio was then calculated from the ratio of the amounts of substance of the two elements:

(4) $$molar\;ratio\;\;\left(S\;:Au\right)=\frac{1.53\;\cdot 10^{-5}\;\mathrm{mol}}{2.82\;\cdot 10^{-5}\;\mathrm{mol}}=0.54\;\left(S\right)\;:1\;\left(Au\right)$$

A average particle diameter of 2 nm was assumed for the number of atoms per particle, based on the results of the TEM (metal core). This gives about 250 gold atoms per nanoparticle as reported earlier.^[^ ^14^ ^]^

The number of ligands per nanoparticle then results from the number of atoms and the molar ratio of sulfur to gold:

(5) DDTperNP=molarratio·atomsperNP=0.54·250=135

The molecular footprint can be determined from the particle surface *A*_NP_ and the number of ligands per particle:

(6) $$molecular\;footprint=\frac{A_{\mathrm{NP}}}{DDT\;per\;NP}=\frac{4\pi r{\left(NP\right)}^{2}}{DDT\;per\;NP}\;$$

### Methods and instruments

DCS was performed with a CPS Instruments DC 24,000 disc centrifuge at 24,000 rpm. A density gradient was prepared with two different sucrose solutions (8 and 24 wt%). To prevent evaporation, 0.5 mL dodecane were used. Polyvinylchloride (PVC) in water with a hydrodynamic diameter of 483 nm was used as standard, provided by CPS Instruments.

TEM measurements were performed with an aberration‐corrected FEI Titan transmission electron microscope with a Cs‐probe corrector (CEOS Company) at a voltage of 300 kV.^[^ ^53^ ^]^ The images were analyzed with the program ANTEMA.^[^ ^54^ ^]^ UV‐Vis spectroscopy was performed on a Genesis 50 instrument (Thermo Scientific) in a quartz cuvette (600 µL) from 200 to 800 nm. The gold concentration was determined by atomic absorption spectroscopy (AAS) with a Thermo Electron M‐Series spectrometer (graphite tube furnace; operated according to DIN EN ISO/IEC 17 025:2005). For sample preparation, 10 µL of the sample was dissolved in 990 µL *aqua regia* and then diluted with 4 mL water. ICP‐MS was used to determine gold, sulfur, and nitrogen with a Spectro Model Spectro Arcos instrument after microwave digestion at Microanalytical Laboratory Kolbe (Fraunhofer Institut Umsicht, Oberhausen). NMR spectroscopy was carried out with a Bruker Avance Neo 400 MHz spectrometer. The freeze‐dried particles were dispersed in 600 µL toluene‐d_8_. IR spectra were obtained from a Jasco FT/IR‐4600 instrument. Mass spectra were obtained with a Bruker maXis 4G instrument with electrospray ionization (Supporting Information). For elemental analysis (CHNS), an EURO EA elemental analyzer (EURO VECTOR) was used. Photoswitching UV‐Vis experiments were conducted with a Jasco V‐550 spectrometer in quartz cuvettes (2 mL) from 250 to 700 nm. Irradiation of the samples was carried out with different LEDs combined with a power supply (12 V, 700 mA): 365 nm (OSRAM, 1.36 W), 405 nm (EDISON, 0.67 W), 460 nm (OSRAM, 0.85 W), and 520 nm (Cree, 87.4 lm). POM was performed on untreated glass slides on a Nikon Eclipse Ni microscope, equipped with a Linkham LTS420 heating stage with crossed polarizers. Micrographs were recorded on an OptixCam Summit K2 OCS‐D3K4‐14 camera in the second heating cycle. Irradiation of the liquid crystalline samples was performed with a 365 nm Hönle bluepoint LED eco at 35% of maximum capacity (20 W cm^−2^). Dynamic scanning calorimetry (DSC) was performed with a Mettler Toledo DSC 3+ Star^e^ System under nitrogen atmosphere. The liquid crystalline phase (3 to 5 mg) was weighed into a 40 µL standard aluminum crucicle and heated and cooled at 10 K min^−1^ between 0 and 100 °C in three consecutive cycles. This measurement was performed in triplicate.

### DOSY‐NMR spectroscopy

DOSY‐NMR spectroscopy of dispersed nanoparticles was performed on a 700 MHz Bruker NEO spectrometer with a 5 mm TCI cryoprobe with *z*‐gradient at 25 °C. The freeze‐dried particles (4 mg) were dispersed in 200 µL toluene‐d_8_. Spectra were measured with a diffusion time of *Δ  =  *100 ms and a pulsed gradient duration of *δ  =  *3.5 ms. The gradient strength was incremented from 5 to 95% of the maximum gradient strength (64.2 G cm^−1^ for a smoothed square gradient pulse) in 32 linear steps. Spectra were processed with the program Topspin 4.4 (Bruker). The linearized diffusion data were plotted and fitted according to the Stejskal–Tanner equation:^[^ ^43^, ^55^ ^]^

(7) $$\ln\left(\frac{I}{I_{0}}\right)=-\gamma^{2}\delta^{2}\left(\Delta -\delta /3\right)\;D\;G^{2}$$

with *I * =  signal intensity, *I*_0_  =  signal intensity without gradient, *γ  =* gyromagnetic ratio of ^1^H, *δ  =  *diffusion gradient pulse length, Δ*  =  *diffusion delay, *G*  = gradient strength, and *D*  = translational diffusion coefficient.

The linearized relative intensities for all nanoparticle signals were averaged. The error bars represent the standard deviation of these signals. While the standard error of the Stejskal‐Tanner fit itself is small (<2%), we estimate the error of the diffusion coefficient to 20% due to manual integration and potentially overlaying small signals from impurities.

The hydrodynamic diameter was calculated according to the Stokes‐Einstein equation:^[^ ^56^ ^]^

(8) $$d_{H}=\frac{k_{B}T}{3\pi \eta D}$$

with *d*_H_  =  hydrodynamic diameter, *k*_B_  = Boltzmann constant, *T*  = temperature in K, *η*  = dynamic viscosity at 25 °C, and *D*  =  translational diffusion coefficient.

## Conflict of Interests

The authors declare no conflict of interest.

## Supporting information



Supporting Information

## Data Availability

All primary data generated or analyzed during this study are included in article and the corresponding supplementary information.

## References

[1] M. Epple , V. M. Rotello , K. Dawson , Acc. Chem. Res. 2023, 56, 3369. 37966025 10.1021/acs.accounts.3c00459 PMC12670212

[2] L. S. Wagner , O. Prymak , T. Schaller , C. Beuck , K. Loza , F. Niemeyer , N. Gumbiowski , K. Kostka , P. Bayer , M. Heggen , C. L. P. Oliveira , M. Epple , J. Phys. Chem. B 2024, 128, 4266. 38640461 10.1021/acs.jpcb.4c01294

[3] K. Klein , M. Hayduk , S. Kollenda , M. Schmiedtchen , J. Voskuhl , M. Epple , Molecules 2022, 27, 1788. 35335152 10.3390/molecules27061788 PMC8949416

[4] S. B. van der Meer , T. Seiler , C. Buchmann , G. Partalidou , S. Boden , K. Loza , M. Heggen , J. Linders , O. Prymak , C. L. P. Oliveira , L. Hartmann , M. Epple , Chem. Eur. J. 2021, 27, 1451. 32959929 10.1002/chem.202003804 PMC7898849

[5] T. Ruks , K. Loza , M. Heggen , O. Prymak , A. L. Sehnem , C. L. P. Oliveira , P. Bayer , C. Beuck , M. Epple , ACS Appl. Bio Mater. 2021, 4, 945.

[6] T. Ruks , K. Loza , M. Heggen , C. Ottmann , P. Bayer , C. Beuck , M. Epple , ChemBioChem 2021, 22, 1456. 33275809 10.1002/cbic.202000761 PMC8248332

[7] Y. Li , R. Juarez‐Mosqueda , Y. Song , Y. Zhang , J. Chai , G. Mpourmpakis , R. Jin , Nanoscale 2020, 12, 9423. 32323691 10.1039/d0nr01430c

[8] R. Dinkel , W. Peukert , B. Braunschweig , J. Phys. Condensed Matter 2017, 29, 133002. 28198355 10.1088/1361-648X/aa5a3c

[9] R. Dinkel , B. Braunschweig , W. Peukert , J. Phys. Chem. C 2016, 120, 1673.

[10] A. M. Smith , L. E. Marbella , K. A. Johnston , M. J. Hartmann , S. E. Crawford , L. M. Kozycz , D. S. Seferos , J. E. Millstone , Anal. Chem. 2015, 87, 2771. 25658511 10.1021/ac504081k

[11] N. Wolff , N. Białas , K. Loza , M. Heggen , T. Schaller , F. Niemeyer , C. Weidenthaler , C. Beuck , P. Bayer , O. Prymak , C. L. P. Oliveira , M. Epple , Materials 2024, 17, 3702. 39124365 10.3390/ma17153702 PMC11313250

[12] N. Wolff , S. Kollenda , K. Klein , K. Loza , M. Heggen , L. Brochhagen , O. Witzke , A. Krawczyk , I. Hilger , M. Epple , Nanoscale Adv 2022, 4, 4502. 36341304 10.1039/d2na00250g PMC9595109

[13] S. B. van der Meer , I. Hadrovic , A. Meiners , K. Loza , M. Heggen , S. K. Knauer , P. Bayer , T. Schrader , C. Beuck , M. Epple , J. Phys. Chem. B 2021, 125, 115. 33356267 10.1021/acs.jpcb.0c09846

[14] K. Klein , K. Loza , M. Heggen , M. Epple , ChemNanoMat 2021, 7, 1330.

[15] L. Stricker , E. C. Fritz , M. Peterlechner , N. L. Doltsinis , B. J. Ravoo , J. Am. Chem. Soc. 2016, 138, 4547. 26972671 10.1021/jacs.6b00484

[16] L. Beaute , N. McClenaghan , S. Lecommandoux , Adv. Drug Delivery Rev. 2019, 138, 148. 10.1016/j.addr.2018.12.010 30553952

[17] L. Sansalone , S. Tang , Y. Zhang , E. R. Thapaliya , F. M. Raymo , J. Garcia‐Amoros , Top. Curr. Chem. 2016, 374, 73. 10.1007/s41061-016-0073-8 27683098

[18] Z. Tian , W. Wu , A. D. Li , ChemPhysChem 2009, 10, 2577. 19746389 10.1002/cphc.200900492

[19] Y. Zhang , K. Zhang , J. Wang , Z. Tian , A. D. Li , Nanoscale 2015, 7, 19342. 26445313 10.1039/c5nr05436b

[20] W. Zhong , L. Shang , Chem. Sci. 2024, 15, 6218. 38699274 10.1039/d4sc00114a PMC11062085

[21] D. Manna , T. Udayabhaskararao , H. Zhao , R. Klajn , Angew. Chem. Int. Ed. Engl. 2015, 54, 12394. 25959725 10.1002/anie.201502419

[22] R. Klajn , K. J. Bishop , B. A. Grzybowski , Proc. Natl. Acad. Sci. USA 2007, 104, 10305. 17563381 10.1073/pnas.0611371104 PMC1965508

[23] K. Zarschler , L. Rocks , N. Licciardello , L. Boselli , E. Polo , K. P. Garcia , L. De Cola , H. Stephan , K. A. Dawson , Nanomedicine 2016, 12, 1663. 27013135 10.1016/j.nano.2016.02.019

[24] H. Häkkinen , Nat. Chem. 2012, 4, 443. 22614378 10.1038/nchem.1352

[25] L. E. Marbella , J. E. Millstone , Chem. Mater. 2015, 27, 2721.

[26] G. Salassa , T. Burgi , Nanoscale Horiz. 2018, 3, 457. 32254134 10.1039/c8nh00058a

[27] N. Wolff , C. Beuck , T. Schaller , M. Epple , Nanoscale Adv. 2024, 6, 3285. 38933863 10.1039/d4na00139g PMC11197423

[28] M. Baginski , M. Tupikowska , G. Gonzalez‐Rubio , M. Wojcik , W. Lewandowski , Adv. Mater. 2020, 32, e1904581. 31729083 10.1002/adma.201904581

[29] N. Brouckaert , N. Podoliak , T. Orlova , D. Bankova , A. F. De Fazio , A. G. Kanaras , O. Hovorka , G. D'Alessandro , M. Kaczmarek , Nanomaterials 2022, 12, 341. 35159688 10.3390/nano12030341 PMC8839905

[30] D. N. Chausov , A. D. Kurilov , R. N. Kucherov , A. V. Simakin , S. V. Gudkov , J. Phys. Condens. Matter 2020, 32, 395102. 10.1088/1361-648X/ab966c 32454469

[31] A. Lal , H. Verma , S. Chirra , R. Dhar , R. Dabrowski , K. L. Pandey , ACS Omega 2023, 8, 29012–29024. 37599970 10.1021/acsomega.3c01863 PMC10433484

[32] Z. Mai , Y. Yuan , J. B. Tai , B. Senyuk , B. Liu , H. Li , Y. Wang , G. Zhou , I. I. Smalyukh , Adv. Sci. 2021, 8, e2102854. 10.1002/advs.202102854 PMC8596134 34541830

[33] O. H. Piñeres‐Quinones , M. K. Onate‐Socarras , F. Wang , D. M. Lynn , C. Acevedo‐Velez , Langmuir 2024, 40, 3923. 10.1021/acs.langmuir.3c03940 38320298

[34] S. Wang , C. Fu , G. Sun , M. A. Gharbi , C. S. Yelleswarapu , Nanotechnology 2023, 34, 36. 10.1088/1361-6528/acdc2b 37285825

[35] M. Brust , J. Fink , D. Bethell , D. J. Schiffrin , C. Kiely , Chem. Commun. 1995, 1655.

[36] S. R. K. Perala , S. Kumar , Langmuir 2013, 29, 9863. 23848382 10.1021/la401604q

[37] H. Fissan , S. Ristig , H. Kaminski , C. Asbach , M. Epple , Anal. Meth. 2014, 6, 7324. 10.1039/c4ay01203h 40883942

[38] N. Wolff , K. Loza , M. Heggen , T. Schaller , F. Niemeyer , P. Bayer , C. Beuck , C. L. P. Oliveira , O. Prymak , C. Weidenthaler , M. Epple , Inorg. Chem. 2023, 62, 17470. 37820300 10.1021/acs.inorgchem.3c02879

[39] G. H. Woehrle , L. O. Brown , J. E. Hutchison , J. Am. Chem. Soc. 2005, 127, 2172. 15713095 10.1021/ja0457718

[40] M. Wu , A. M. Vartanian , G. Chong , A. K. Pandiakumar , R. J. Hamers , R. Hernandez , C. J. Murphy , J. Am. Chem. Soc. 2019, 141, 4316. 30763078 10.1021/jacs.8b11445

[41] M. J. Hostetler , J. E. Wingate , C. J. Zhong , J. E. Harris , R. W. Vachet , M. R. Clark , J. D. Londono , S. J. Green , J. J. Stokes , G. D. Wignall , G. L. Glish , M. D. Porter , N. D. Evans , R. W. Murray , Langmuir 1998, 14, 17.

[42] K. Salorinne , T. Lahtinen , J. Koivisto , E. Kalenius , M. Nissinen , M. Pettersson , H. Häkkinen , Anal. Chem. 2013, 85, 3489. 23506040 10.1021/ac303665b

[43] E. O. Stejskal , J. E. Tanner , J. Chem. Phys. 1965, 42, 288.

[44] Y. Huang , L. Fuksman , J. Zheng , Dalton Trans. 2018, 47, 6267. 29594274 10.1039/c8dt00420j PMC5940531

[45] V. Amendola , R. Pilot , M. Frasconi , O. M. Marago , M. A. Iati , J. Phys. Condens. Matter 2017, 29, 203002. 28426435 10.1088/1361-648X/aa60f3

[46] H. M. D. Bandara , S. C. Burdette , Chem. Soc. Rev. 2012, 41, 1809. 22008710 10.1039/c1cs15179g

[47] G. W. Gray , K. J. Harrison , J. A. Nash , Electron.Lett 1973, 9, 130.

[48] H. Q. Tran , S. Kawano , R. E. Thielemann , K. Tanaka , B. J. Ravoo , Chem. ‐ Eur. J. 2024, 30, e202302958. 37944022 10.1002/chem.202302958

[49] R. Bonomi , A. Cazzolaro , L. J. Prins , Chem. Commun. 2011, 47, 445. 10.1039/c0cc02260h 20886126

[50] F. Chen , Y. K. Tian , Y. Chen , Chem. Asian J. 2018, 13, 3169. 30284398 10.1002/asia.201801235

[51] F. Malotke , T. Thiele , J. S. Gutmann , M. Giese , ACS Appl. Mater. Interfaces 2023, 15, 53096. 10.1021/acsami.3c10684 37917042

[52] L. M. Liz‐Marzan , Chem. Commun. 2013, 49, 16. 10.1039/c2cc35720h 23032158

[53] A. Thust , J. Barthel , K. Tillmann , J. Large‐scale Res. Fac. 2016, 2, A41.

[54] N. Gumbiowski , K. Loza , M. Heggen , M. Epple , Nanoscale Adv. 2023, 5, 2318. 37056630 10.1039/d2na00781a PMC10089082

[55] A. S. Altieri , D. P. Hinton , R. A. Byrd , J. Am. Chem. Soc. 1995, 117, 7566.

[56] A. Einstein , Ann. Phys 1905, 322, 549.

