# Supplementary material for: Ultrasmall Gold Nanoparticles (2 nm) Decorated with a High Density of Photochemically Switchable Ligands
Source: Chemistry. 2025 Jun 1;31(36):e202501204. doi: 10.1002/chem.202501204 (PMC12202854; doi:10.1002/chem.202501204)
Supplement: Supplementary file 1 — Supporting Information [file CHEM-31-e202501204-s001.pdf]

Supporting Information

Ultrasmall Gold Nanoparticles (2 nm) Decorated with a High Density of Photochemically Switchable Ligands

Lisa-Sofie Wagner,[1,+] Tobias Thiele,[2,+] Kateryna Loza,[1] Christine Beuck,[3] Peter Bayer,[3] Marc Heggen,[4] Michael Giese[2,*] and Matthias Epple[1,*]

Supporting Information

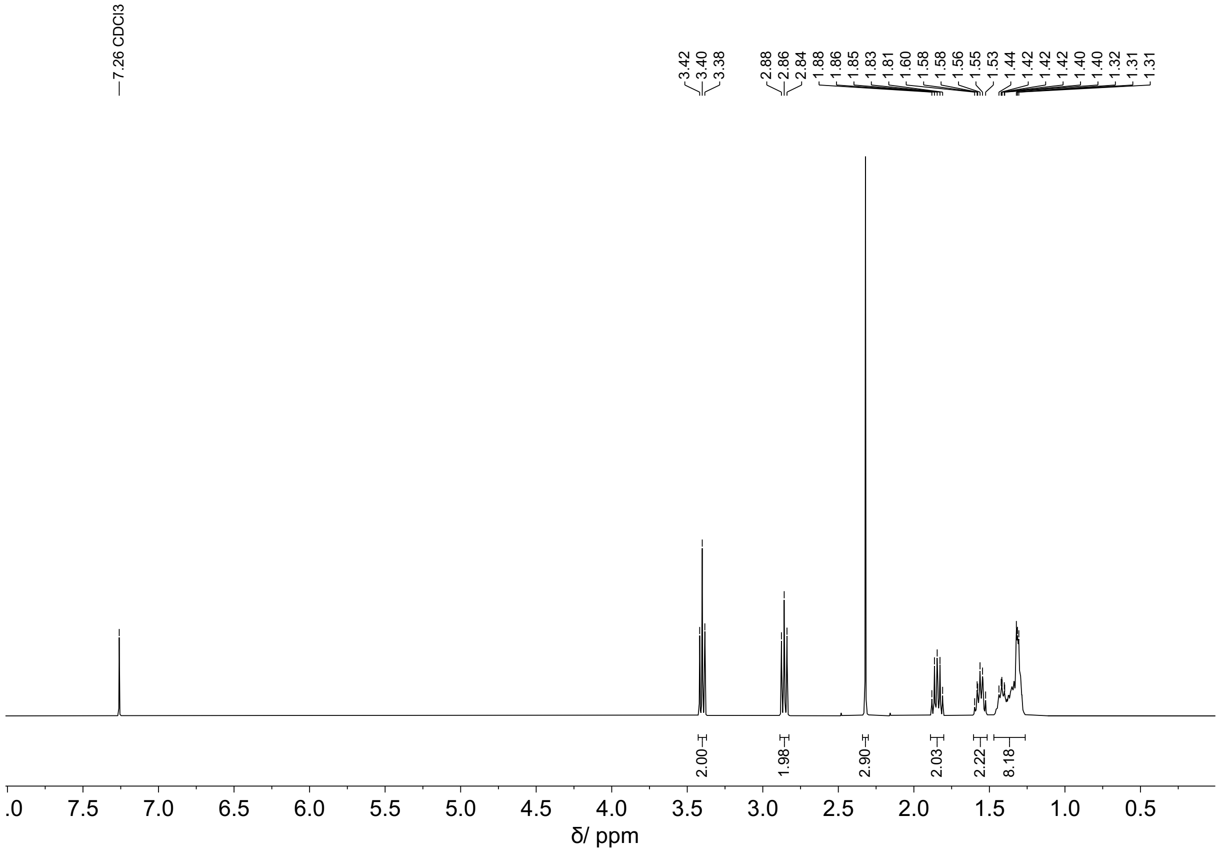

Figure S1: ^{1}H-NMR spectrum of alkylation agent **1** in $CDCl_3$ (400 MHz, 298 K).

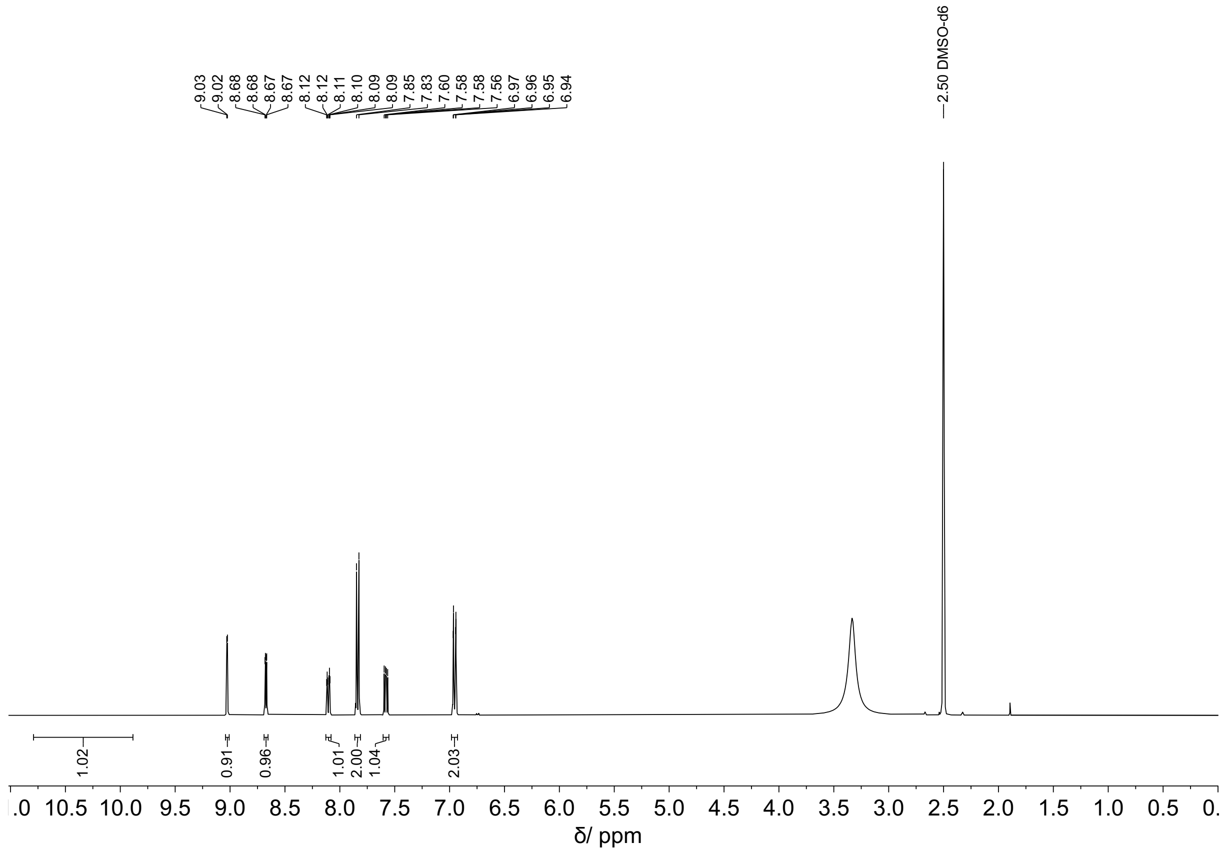

Figure S2: ^{1}H-NMR spectrum of azo compound **2** in DMSO-d_6 (400 MHz, 298 K).

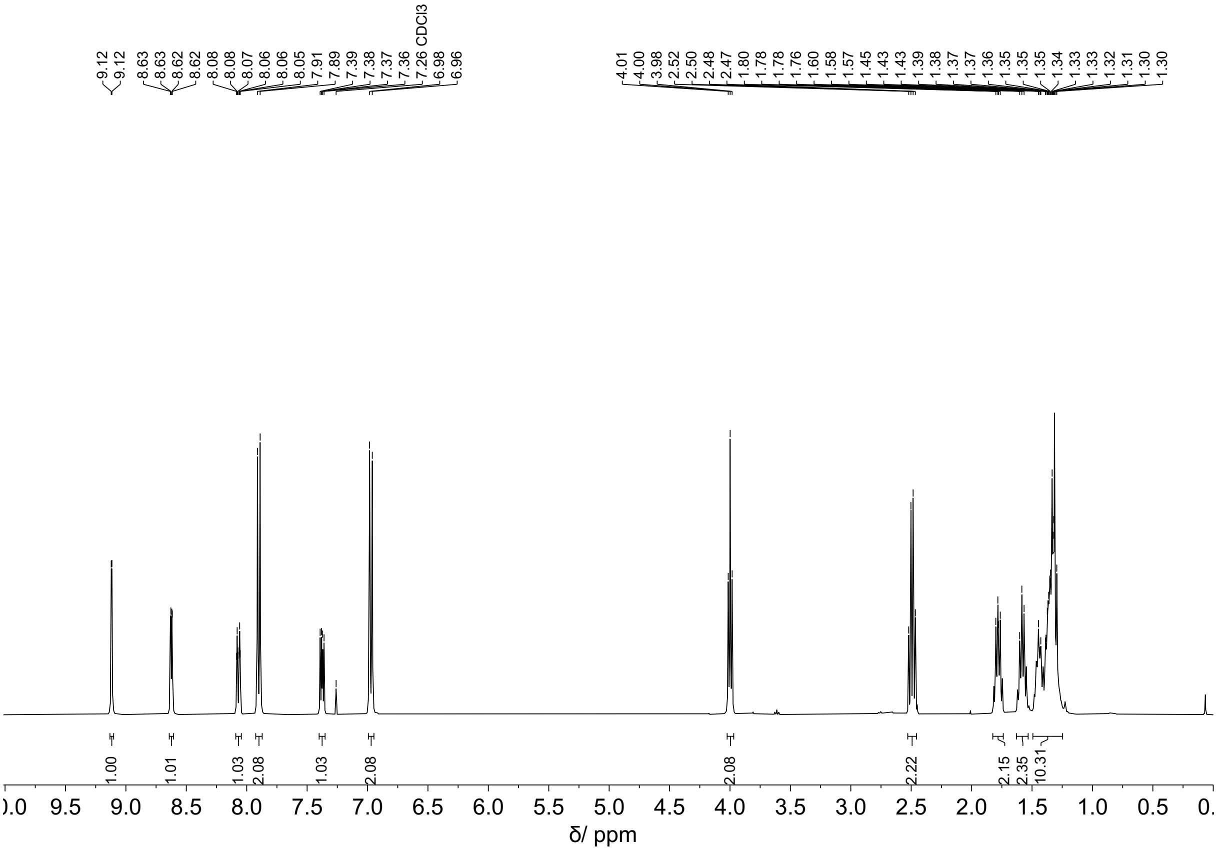

Figure S3: ^{1}H-NMR spectrum of ligand **3** in $CDCl_3$ (400 MHz, 298 K).

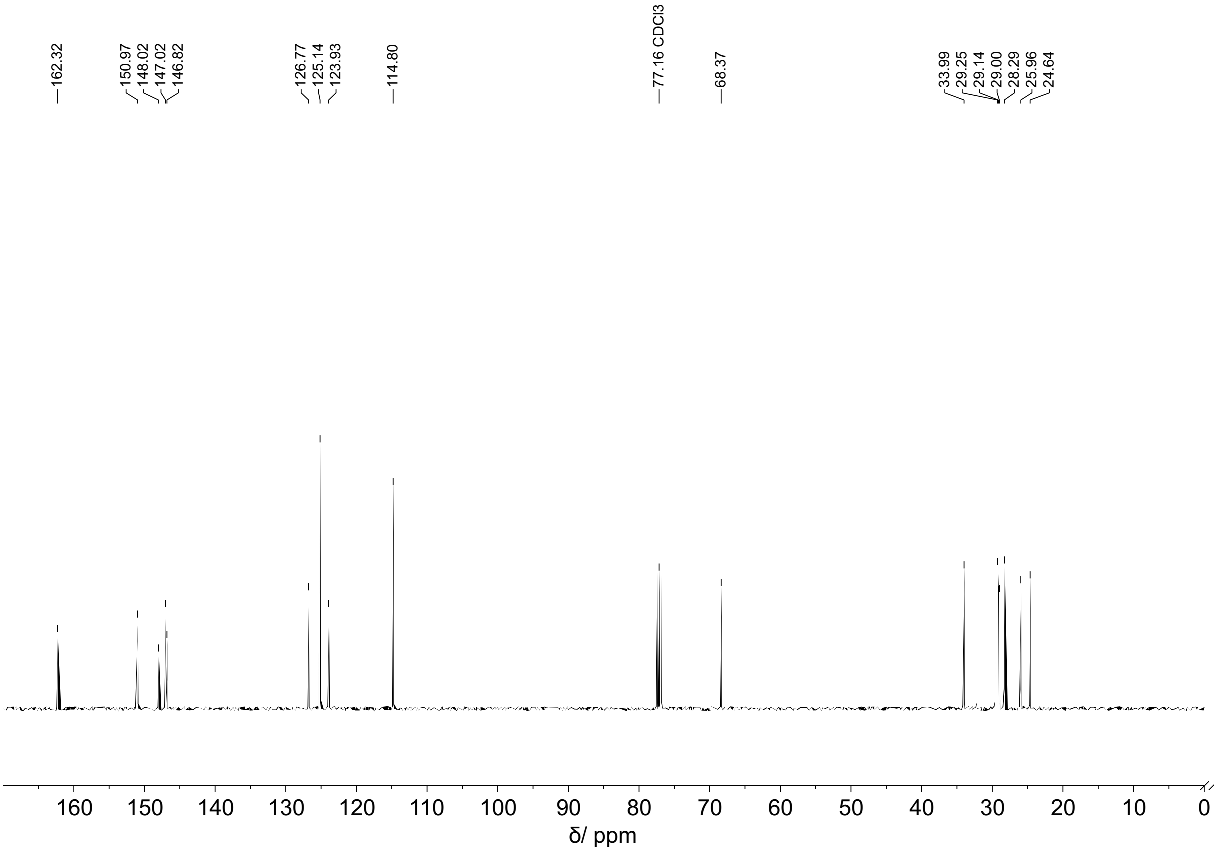

Figure S4: ^{13}C-NMR spectrum of ligand **3** in $CDCl_3$ (101 MHz, 298 K).

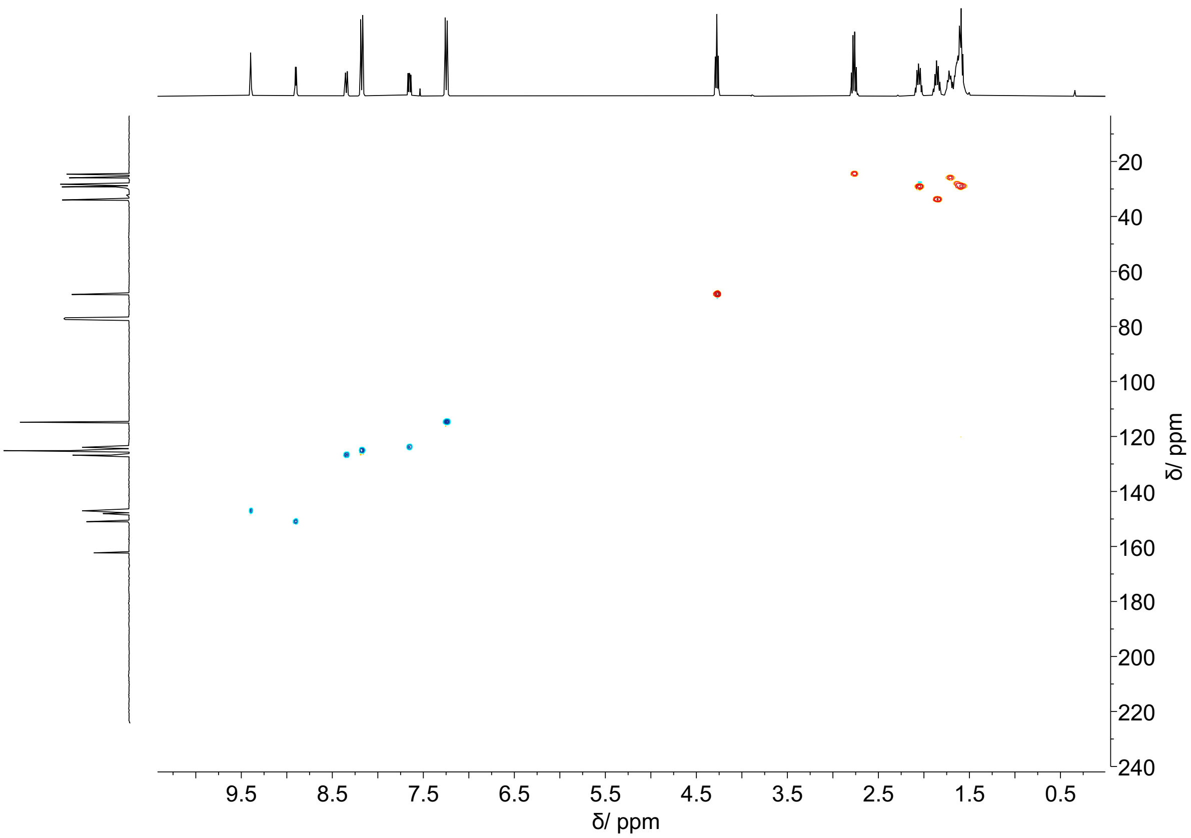

Figure S5: ^{1}H-^{13}C-HSQC spectrum of ligand **3** in $CDCl_3$ (298 K).

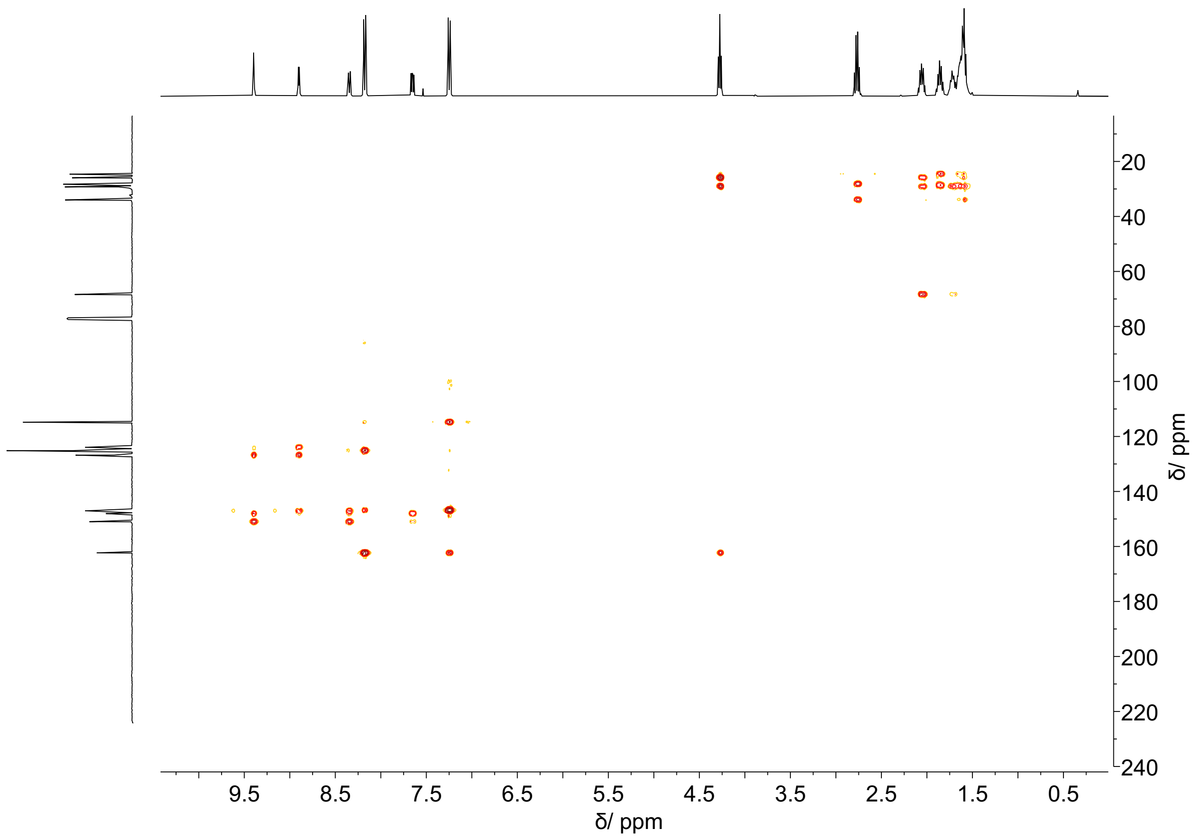

Figure S6: ^{1}H-^{13}C-HMBC spectrum of ligand **3** in $CDCl_3$ (298 K).

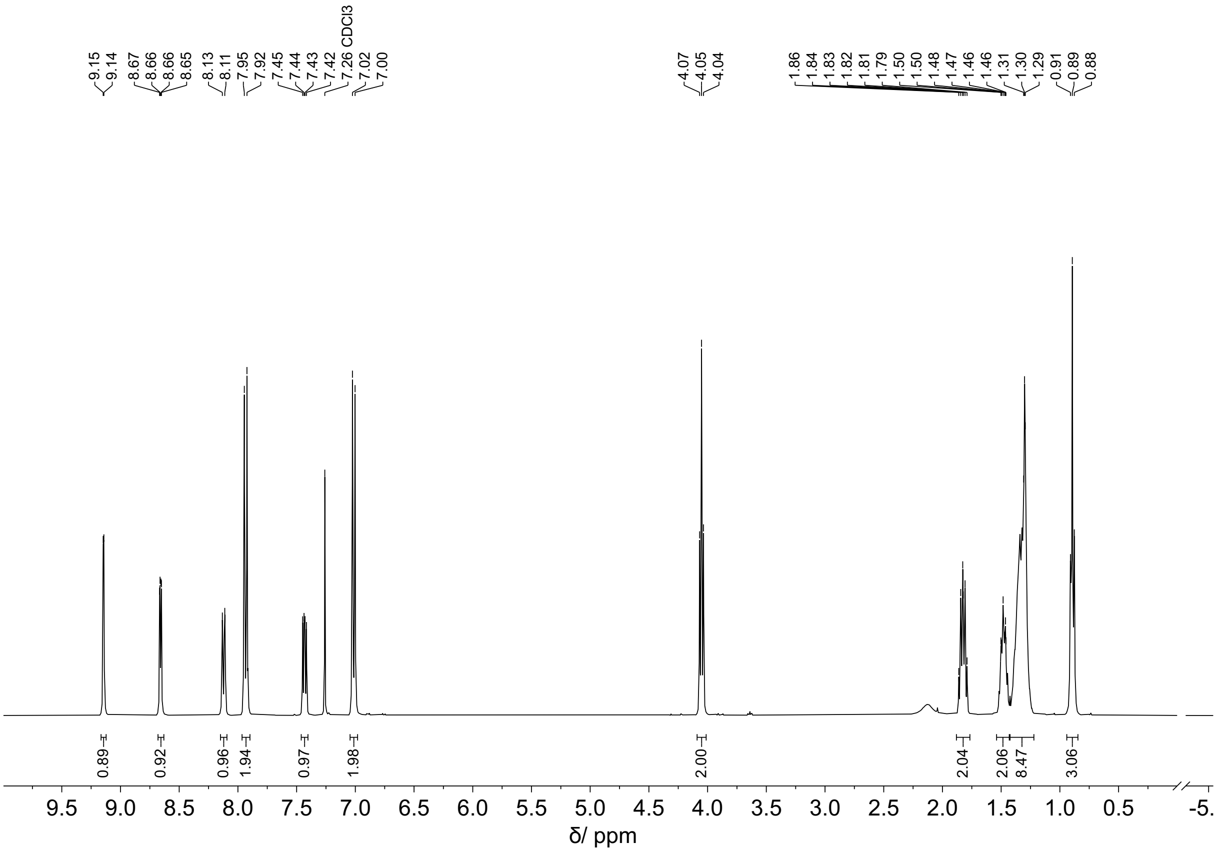

Figure S7: ^{1}H-NMR spectrum of azo compound **4** in $CDCl_3$ (400 MHz, 298 K).

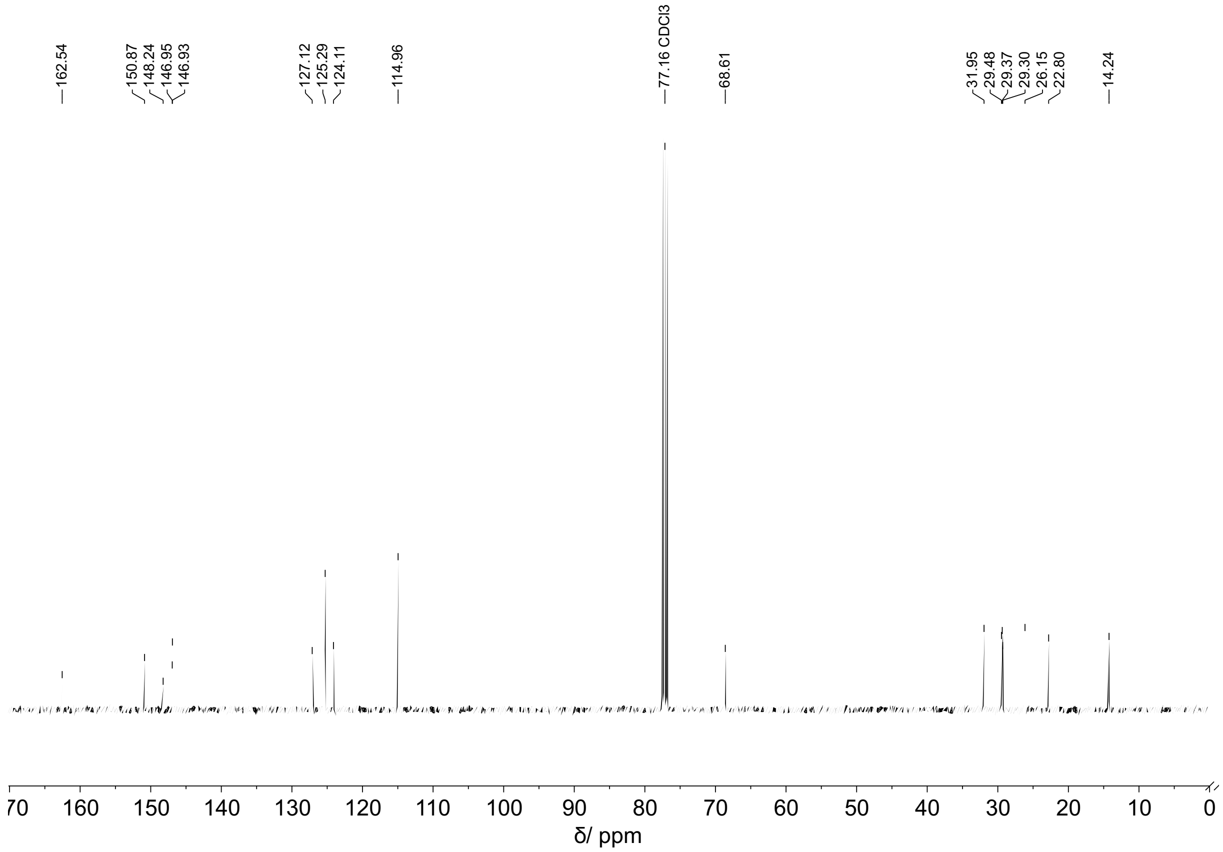

Figure S8: ^{13}C-NMR spectrum of azo compound **4** in $CDCl_3$ (101 MHz, 298 K).

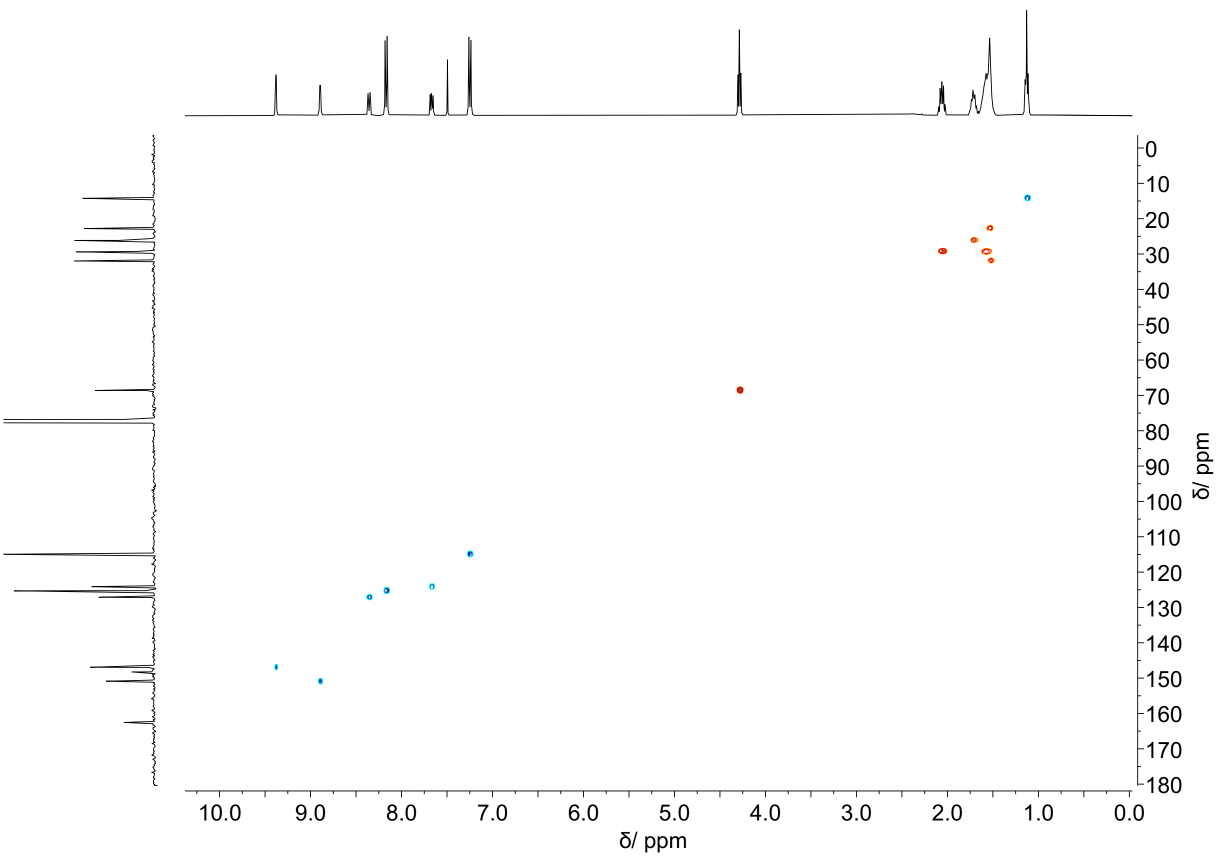

Figure S9: ^{1}H-^{13}C-HSQC spectrum of azo compound **4** in $CDCl_3$ (298 K).

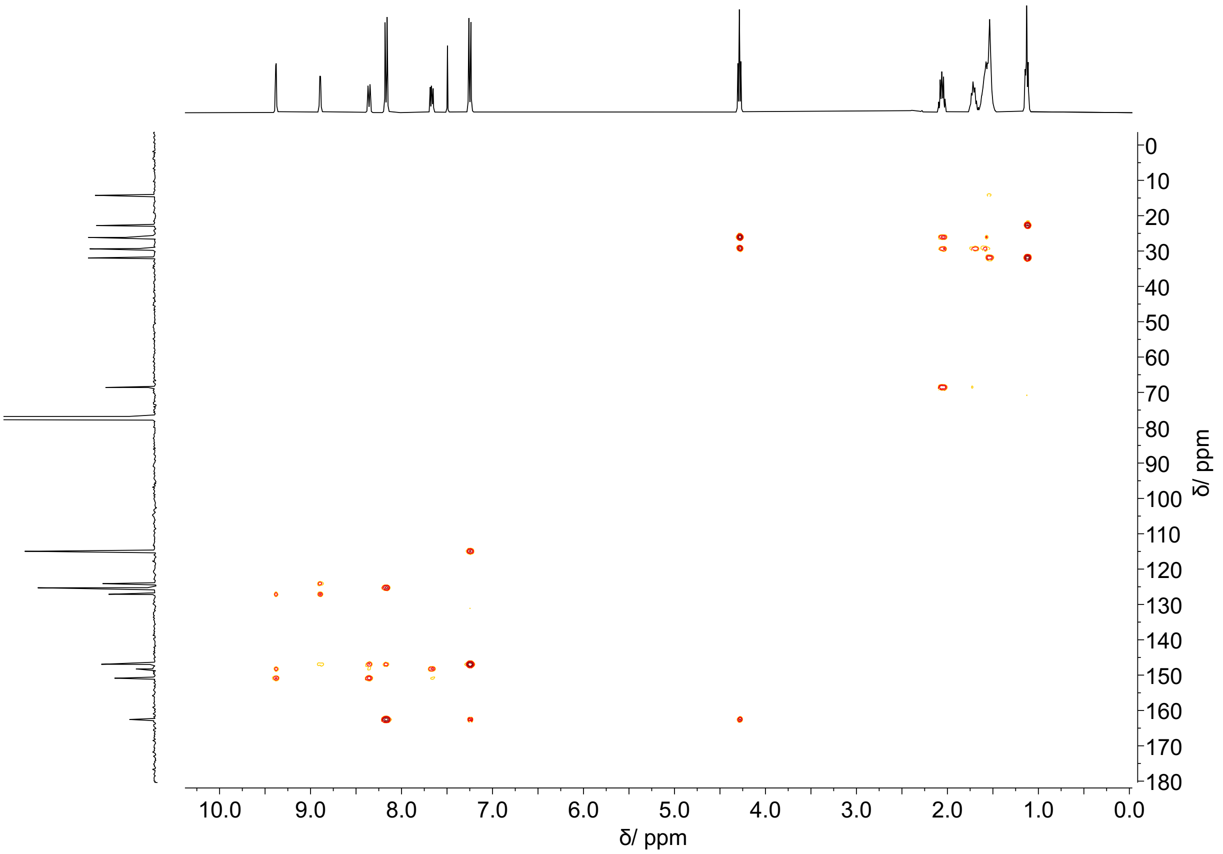

Figure S10: ^{1}H-^{13}C-HMBC spectrum of azo compound **4** in $CDCl_3$ (298 K).

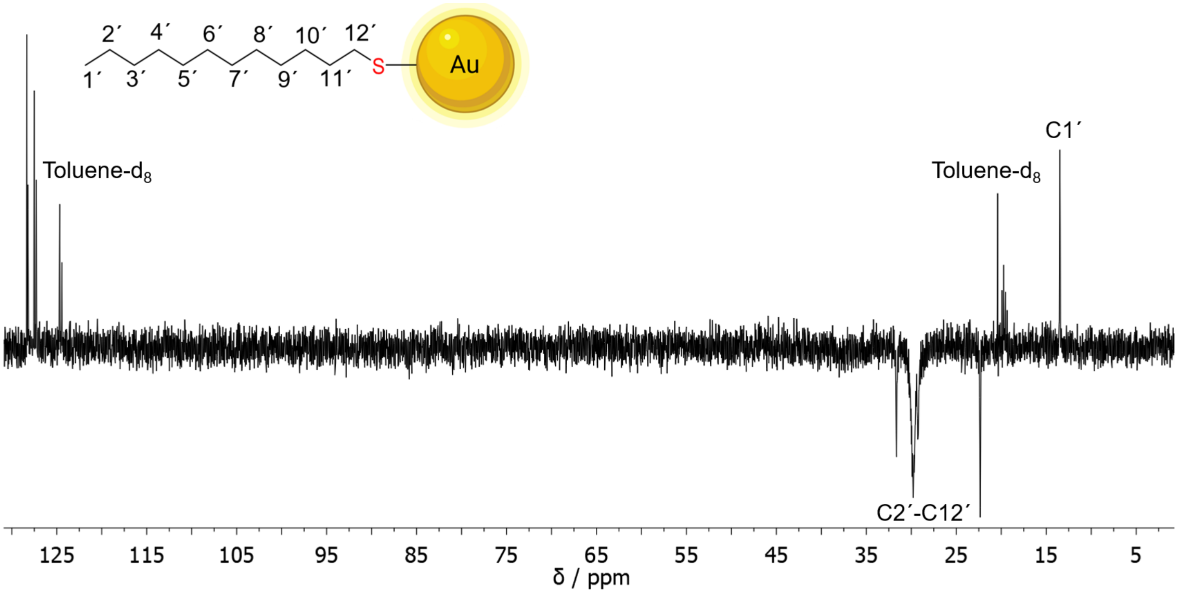

Figure S11: ^{13}C-DEPTQ NMR spectrum of AuDDT nanoparticles, dispersed in toluene-d$_8$ (298 K).

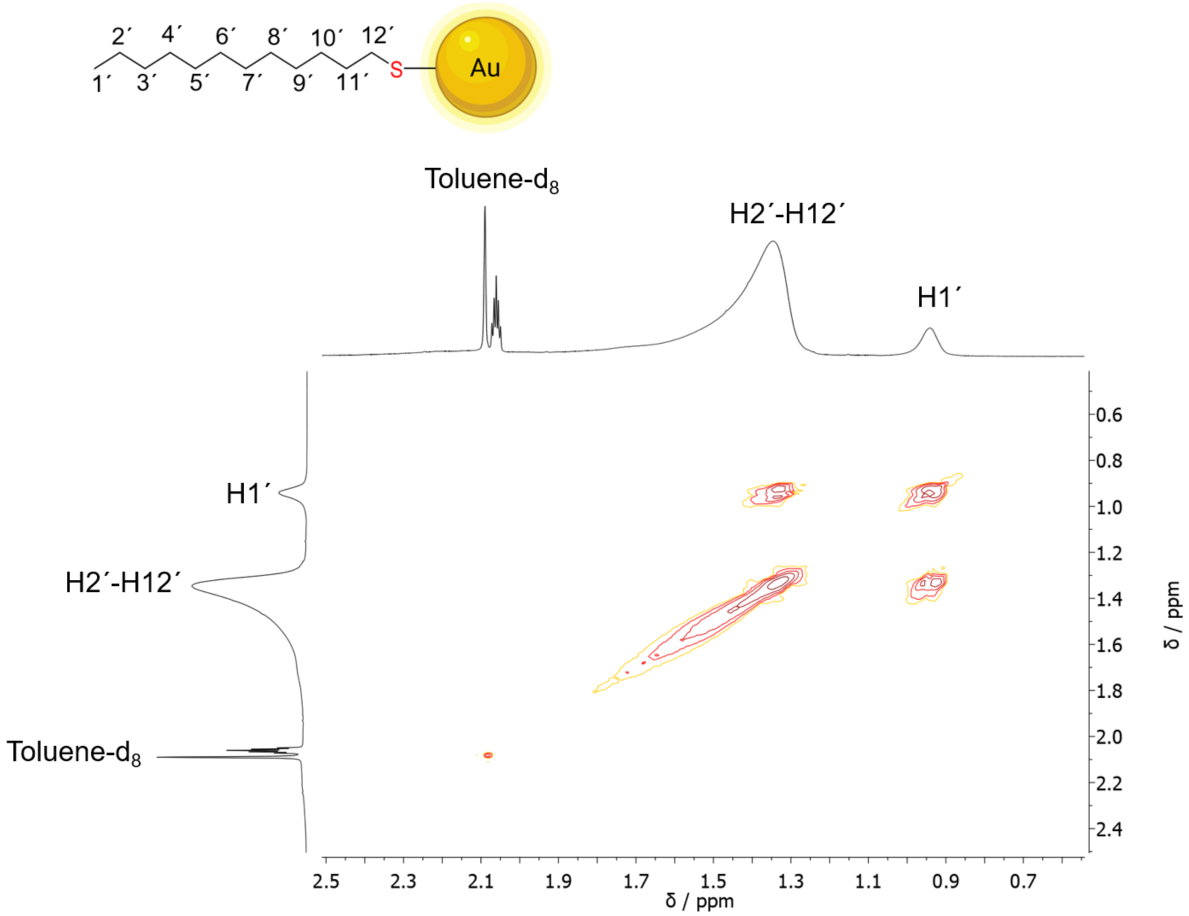

Figure S12: ^{1}H-^{1}H-COSY spectrum of AuDDT nanoparticles, dispersed in toluene-d$_8$ (298 K).

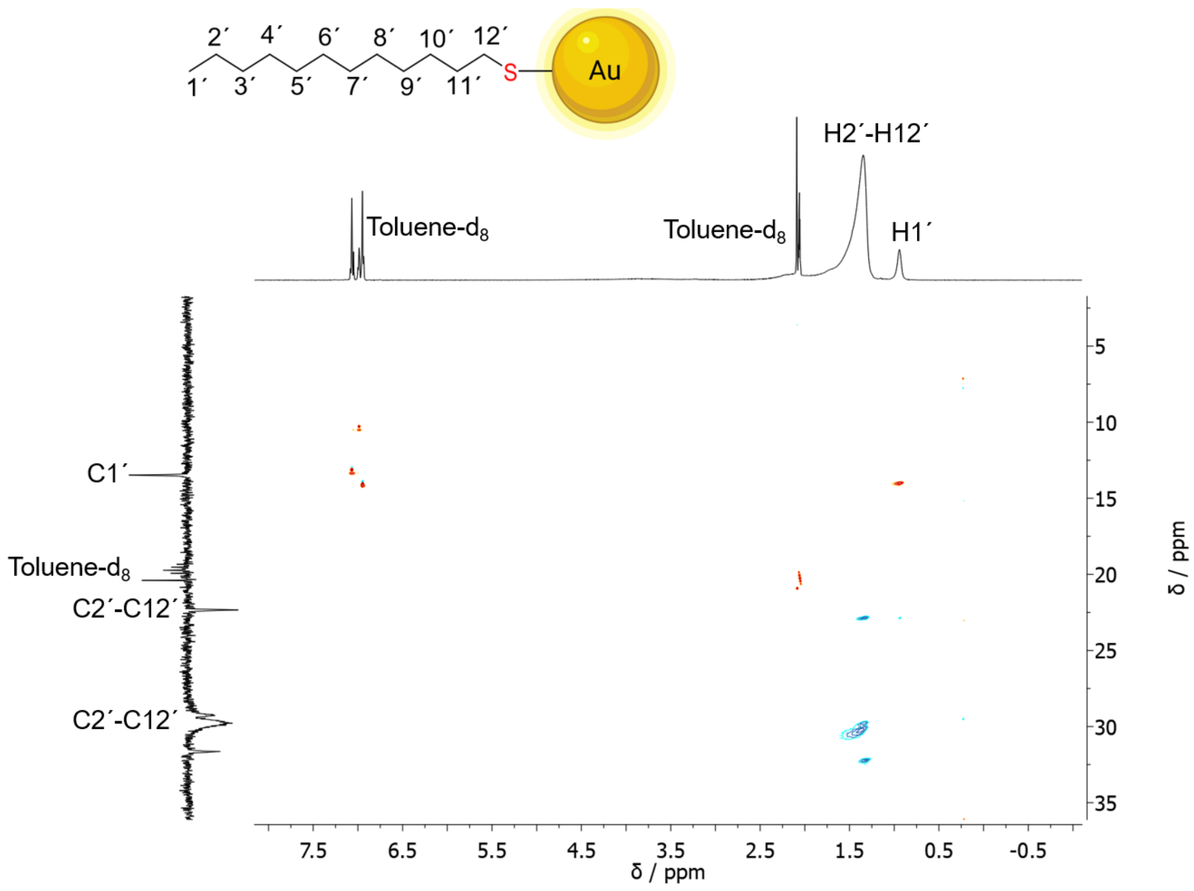

Figure S13: ^{1}H-^{13}C-HSQC spectrum of AuDDT nanoparticles, dispersed in toluene-d₈ (298 K).

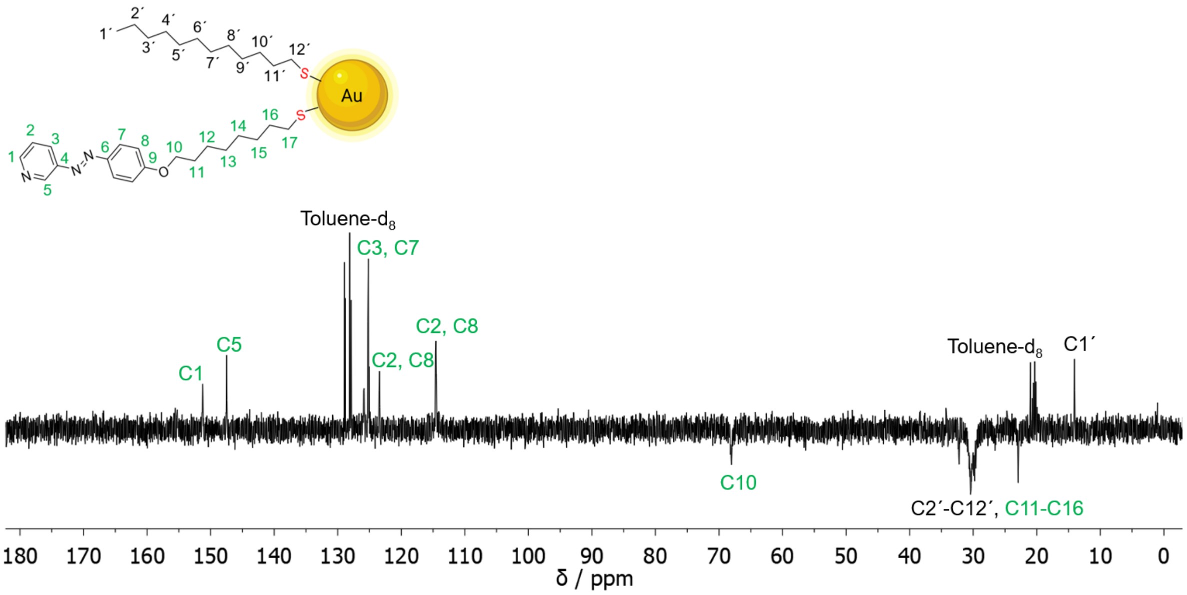

Figure S14: ^{13}C-DEPTQ NMR spectrum of AuAzo nanoparticles, dispersed in toluene-d_8 (298 K).

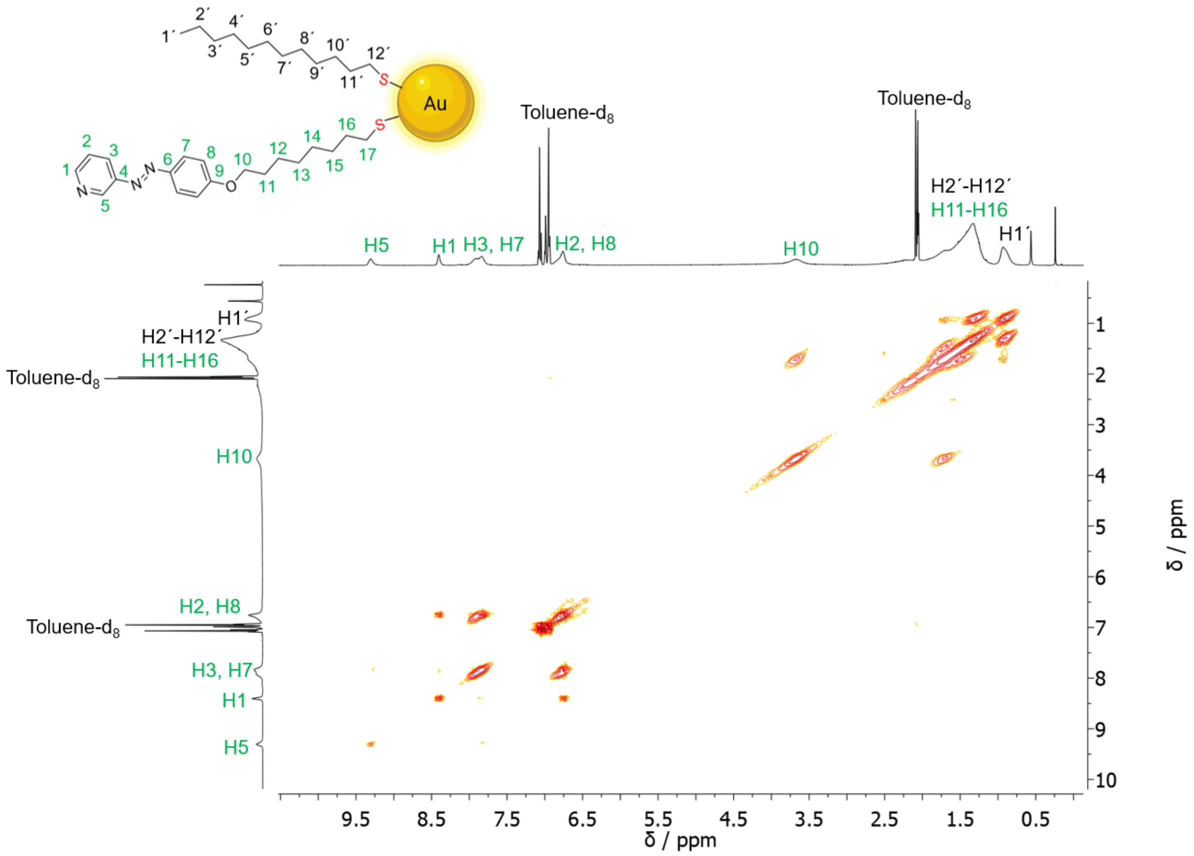

Figure S15: ^{1}H-^{1}H-COSY spectrum of AuAzo nanoparticles, dispersed in toluene-d_8 (298 K).

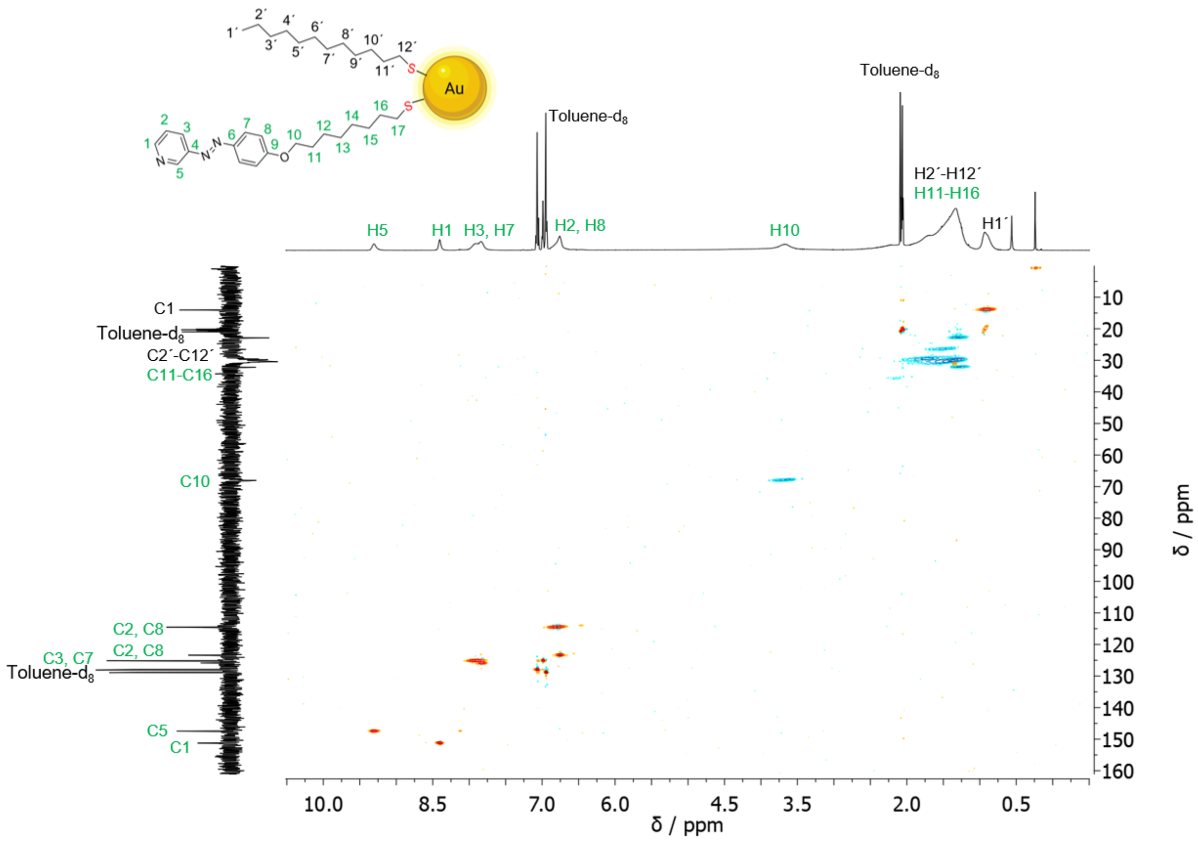

Figure S16: ^{1}H-^{13}C-HSQC spectrum of AuAzo nanoparticles, dispersed in toluene-d_8 (298 K).

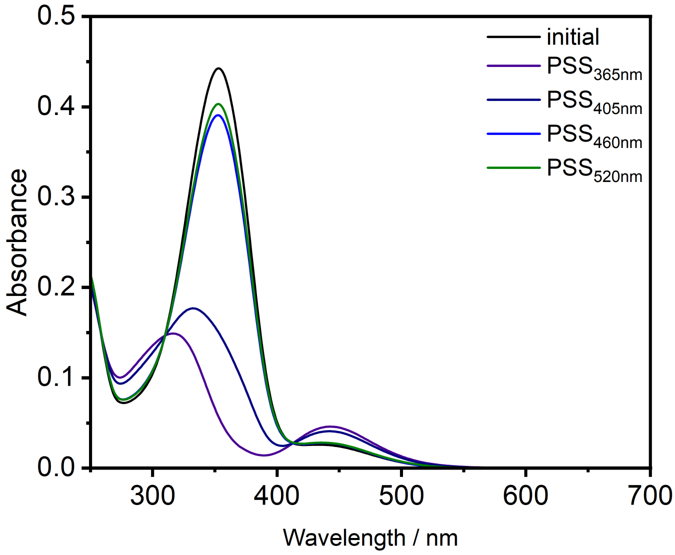

Figure S17: UV-Vis absorption spectra of azo ligand **3** dissolved in DCM (20 μM). **Black**: Initial spectrum before irradiation; **violet**: Spectrum of the photostationary state after irradiation with 365 nm; **dark blue**: PSS of 405 nm; **blue**: PSS of 460 nm; **green**: PSS of 520 nm. UV-Vis photoswitching experiments: Irradiation occurred at 365, 405, 460 and 520 nm, respectively, yielding versatile photostationary states. Given that irradiation at 365 nm gave the best *Z*-rich PSS and irradiation at 520 nm the best *E*-rich PSS, these wavelengths were chosen for all further experiments with AuAzo particles.

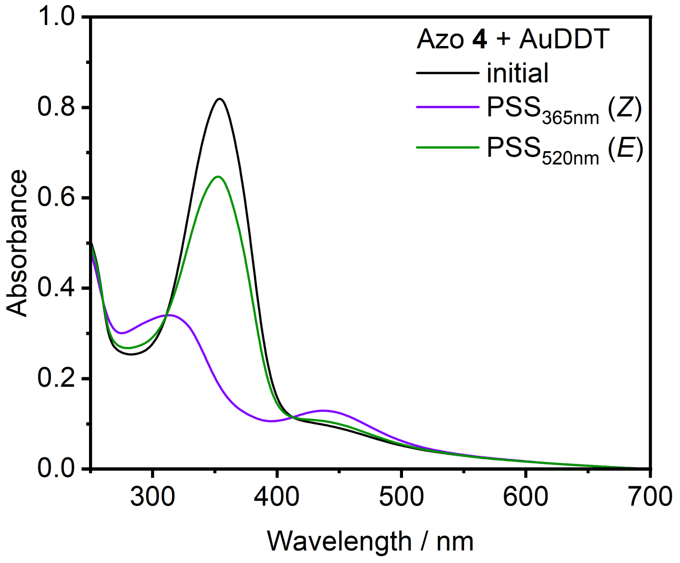

Figure S18: UV-Vis absorption spectra of azo compound **4** with AuDDT in DCM. Azo compound **4** and AuDDT particles were mixed in the same molar ratio of DDT and ligand **3** in AuAzo nanoparticles (1.4 : 1). Azo compound **4** was used instead of ligand **3** to prevent ligand exchange during the experiment. The mixture was diluted until an absorption of about 0.8 was reached. **Black**: Initial spectrum before irradiation; **violet**: Spectrum of the photostationary state after irradiation with 365 nm (3 s); **green**: PSS of 520 nm (60 s). Photostationary states were obtained after similar times of irradiation, indicating that the conjugation of ligand **3** to the nanoparticles does not increase the necessary time to reach the PSS (Fig. 7).

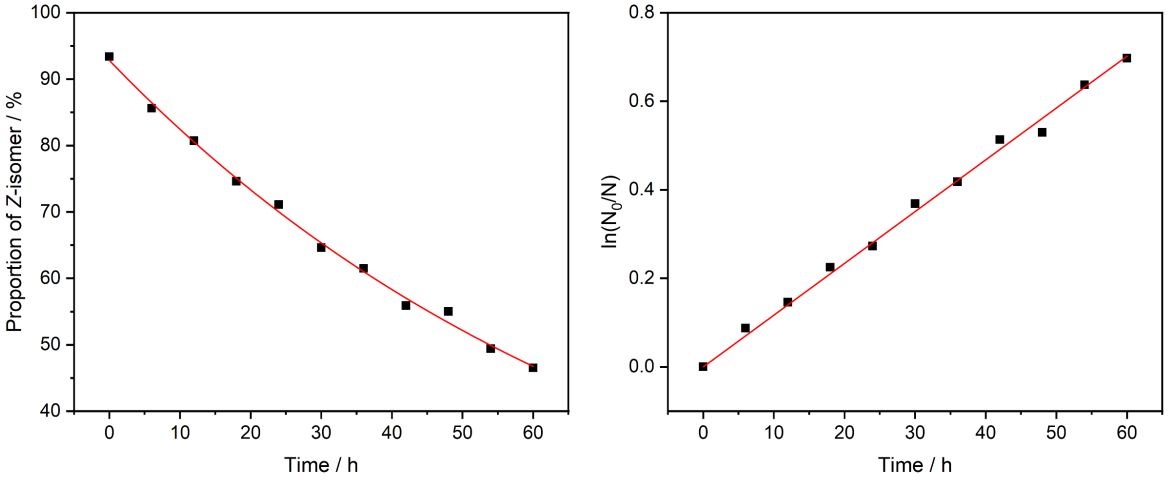

Figure S19: Thermal dark relaxation of ligand **3** dissolved in DCM-d_2 (10 mM). Plot of the proportion of *Z*-isomer versus time (**left**) and its linearization according to a first-order reaction (**right**). The NMR experiments for thermal dark relaxation of ligand **3** in DCM-d2 (10 mM) were conducted as described for the AuAzo particles. An irradiated solution (365 nm) was transferred to a brown glass NMR tube and kept in the dark at ambient temperature, and an ^{1}H-NMR spectrum was taken every 6 h.

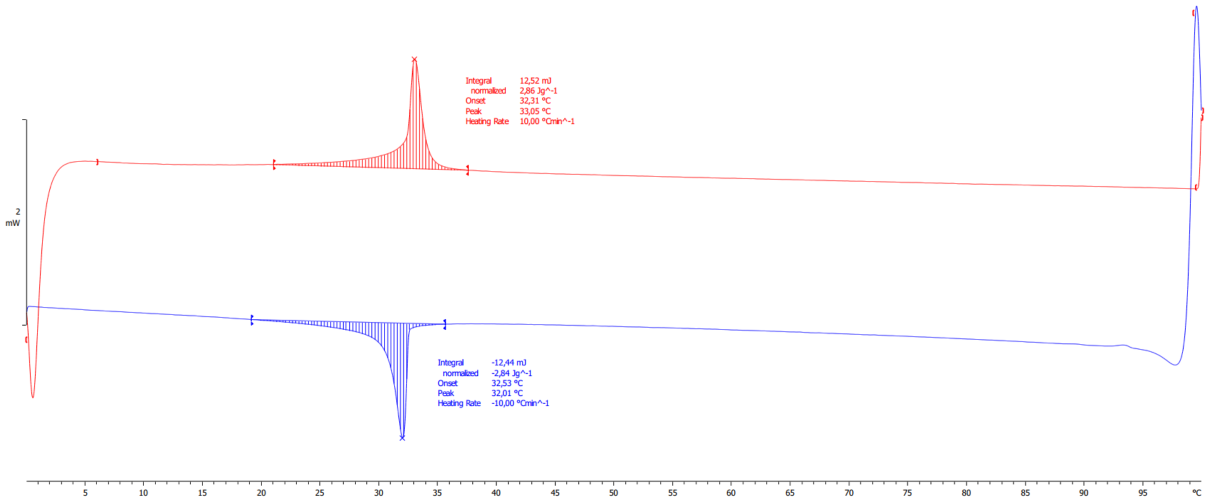

Figure S20: Representative second heating (**red**) and cooling cycle (**blue**) of AuAzo in 5CB (0.5 wt%, endothermal peaks pointing upwards). A heating rate of 10 K min^{-1} and 3 to 5 mg of the liquid crystal were used.
